# Tracking Out-Of-Equilibrium Growth of Bimetallic Nanoalloys Using Deep Learning and X‑ray Scattering

**DOI:** 10.1021/acsnano.6c09310

**Published:** 2026-07-21

**Authors:** Lucia Allara, Georg Daniel Förster, Pascal Andreazza, Federica Bertolotti, Antonietta Guagliardi

**Affiliations:** † Department of Science and High Technology, 19045 University of Insubria, Via Valleggio 11, Como 22100, Italy; ‡ 27047 Université d’Orléans, CNRS, Interfaces, Confinement, Matériaux et Nanostructures (ICMN), Orléans 45071, France; § 567340 Istituto di Cristallografia, Consiglio Nazionale delle Ricerche (CNR), via Valleggio 11, Como 22100, Italy; ∥ Total Scattering Laboratory (To.Sca.Lab), Department of Science and High Technology, University of Insubria, Via Valleggio 11, Como 22100, Italy; ⊥ INSTM, Consorzio Interuniversitario per la Scienza e Tecnologia dei Materiali, Via G. Giusti 9, Firenze 50121, Italy

**Keywords:** nanoalloys, Debye Scattering Equation, X-ray scattering, deep Learning, molecular dynamics

## Abstract

Reconstructing the growth of metallic nanoparticles in real time remains a long-standing challenge, particularly for nanoalloy systems that exhibit complex atomic spatial arrangements and display structures and properties distinct from those of pure metal particles. Here, we present a methodology that integrates Molecular Dynamics and *in silico* wide-angle X-ray scattering signals, which are calculated using the Debye Scattering Equation, with a deep learning regressor, enabling a direct connection between experiments and data-driven structural interpretation. Atomic-scale descriptors of size and composition of bimetallic nanoparticles, as well as elemental ordering spanning from Janus-like to core–shell arrangements, previously accessible primarily through computational methods, are now extracted using X-ray scattering measured patterns as the sole input information. Applying this model, we reconstruct the growth mechanism of Ag–Co nanoparticles formed in ultra high vacuum by Co vapor deposition onto Ag seeds, monitored *in situ* and in real time by grazing incidence wide-angle X-ray scattering and occurring under strongly out-of-equilibrium conditions. This analysis provides evidence that the nanoparticle structural evolution is not governed solely by the amount of Co supplied but instead results from a kinetically controlled evolution pathway, in line with theoretical predictions. Overall, the developed framework provides a strategy that significantly improves the interpretation of nanoalloys growth processes in real time via X-ray scattering data that would otherwise be difficult or even impossible to analyze through conventional fitting approaches. As such, the presented method is inherently compatible with on-the-fly and high-throughput data collection strategies.

## Graphical abstract

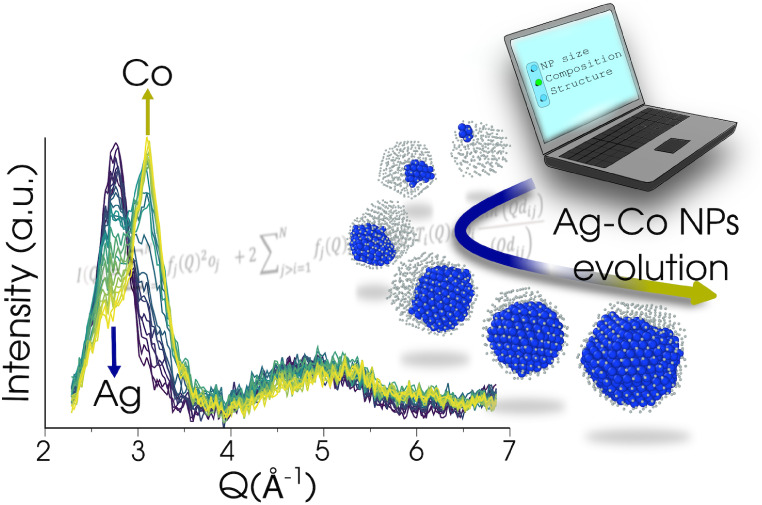

## Introduction

Bimetallic nanoparticles (NPs) are an important class of emerging materials^1^ exhibiting new or exalted properties, due mainly to surface effect and matter confinement, for a wide range of scientific and technological applications,^2–5^ including (electro)­catalysis,^6–13^ electronics,^14–19^ magnetism,^20–22^ photonics,^23–27^ energy storage,^28^ and biomedicine.^29–35^ By integrating two distinct metals into a single cluster, these NPs combine the characteristics of the individual components while also benefiting from new features that arise from synergistic interactions between the metals. For example, architectures combining a catalytic metal (Pd) with a plasmonic one (Au) have shown that antenna-satellite configurations, where a large Au particle enhances the response of a smaller Pd unit, generate hot carriers more efficiently than Au@Pd core–shell structures or isolated Pd nanocrystals.^27^ This case exemplifies the crucial role of spatial arrangement and atomic ordering in bimetallic NPs in determining their properties and potential applications.

The promising physical properties of bimetallic NPs can be accessed and finely tuned by precisely controlling size and shape distributions, composition, and crystal structure during synthesis.^36–40^ Gas-phase syntheses on support enable atomic-level control over nucleation and growth, producing size-selected clusters with quite well-defined composition by accurately adjusting kinetic and thermodynamic parameters as metal(s) deposition rates and metal–support interactions.^41–45^ However, the resulting structural variability is huge, giving rise to many possible configurations. These include chemically ordered and randomly disordered alloys, core–shell structures with single or multiple atomic-layer shells, and segregated Janus architectures. Intermediate arrangements with a continuous structural transition between the latter two idealized limits, such as off-center core–shell, core–satellite, and quasi-Janus configurations, are also observed.^5,36,46^ There is general agreement on the key role played by the metals cohesive energies in determining segregation versus mixing in alloyed clusters. If the heteronuclear (intermetallic) bonds show an energy gain with respect to the homonuclear ones, alloy formation is favored, leading to mixed configurations. Otherwise, segregation is expected, with the metal possessing the higher surface energy, usually linked to higher cohesive energy in metals, occupying the core of the cluster.^46^ More recently, a combined Molecular Dynamics (MD), Monte Carlo, and Principal Components Analysis study,^47^ showed that both the cohesive-energy difference and the Wigner-Seitz radius mismatch jointly determine the degree of segregation in bimetallic systems. Segregated structures such as Janus-like or core–shell configurations emerge only when the cohesive-energy difference exceeds ∼ 20% and the radius mismatch is larger than ∼ 4%, especially when the mixing enthalpy is positive. Otherwise, surface segregation or partial core–shell arrangements primarily depend on the relative abundance of the surface-preferring metal. Moreover, in core–shell systems characterized by a large atomic-size mismatch between core and shell, such as AgCu, AgCo, AgNi, and AuNi nanoalloys, symmetry-breaking shell reconstructions have been identified as energetically favorable. These reconstructions drive a transition from achiral anti-Mackay shells to high-symmetry chiral icosahedral shells, which provide a more optimal balance between strain accommodation and metal–metal bonding than their achiral (Mackay or anti-Mackay) counterparts.^48,49^

This picture is further complicated since bimetallic NPs are often synthesized under out-of-equilibrium conditions, leading to kinetically trapped metastable structures that may evolve over time.^50–52^ As a general trend, thermodynamic products are favored at relatively high synthesis temperatures and low deposition rates. The surface-diffusion rate of the deposited metal further dictates whether growth follows a thermodynamic or kinetic pathway. When surface diffusion is slower than deposition, the kinetically controlled “hit-and-stick” mechanism dominates. Conversely, when diffusion is faster, the “hit-and-diffuse” mechanism prevails, leading to thermodynamically controlled growth.^4,53^

On the experimental side, some attempts have been made to monitor the formation and growth processes of bimetallic NPs during gas-phase synthesis, mostly through spectroscopy techniques, particularly at the earliest stages, where the system consists mainly of gas-phase species or small clusters.^21,36^ However, spectroscopy provides only indirect information on energy levels that must be correlated to structural features, making it difficult to experimentally achieve an atomic-scale reconstruction of the nucleation and growth mechanisms of bimetallic NPs.

On the other hand, wide-angle X-ray scattering (WAXS) methods represent an ideal approach because they provide a direct probe of both structure and microstructure. When applied in total scattering mode (WAXTS) treating both Bragg and diffuse scattering, these techniques offer robust, simultaneous information across multiple length scales, especially in the case of nanocrystalline materials, including size, shape, crystal structure, composition, and morphology.^54–60^ In the field of metallic NPs, both real space (Pair Distribution Function)^59,60^ and reciprocal space (Debye Scattering Equation, DSE)^54,61,62^ WAXTS methods have been successfully applied to characterize a wide variety of systems, from magic-sized clusters with multidomain twinned structures,^40,63–66^ to supported NPs for catalytic applications.^67–70^ In several cases, these methods have also been complemented by unsupervised Machine Learning (ML) approaches, which help reduce manual trial-and-error refinement in favor of a higher degree of automation,^71–78^ and even guide synthetic strategies within the emerging field of autonomous laboratories.^79^ However, these previous studies have been limited to *ex situ* (i.e., steady state or post mortem) data analysis and mainly focused on monometallic systems.

Despite their capabilities, WAXTS analysis remains a time-consuming, customized process,^75,80^ particularly challenging for *in situ* and *operando* experiments now widely accessible in synchrotron facilities that enable fast data acquisition down to the nanosecond time scale. Indeed, these high-throughput data collection strategies are rarely matched by equally quantitative or automated modeling workflows. As a result, data interpretation often remains qualitative, since the modeling step still requires extensive manual exploration of structural candidates that is difficult to reconcile with the terabyte-scale datasets produced in time-resolved experiments.^80–83^

In this work, we developed a workflow designed for the fast retrieval of clusters size (number of atoms), composition, and atomic configuration of bimetallic NPs (spanning from Janus-like to core–shell structures) from *in situ* X-ray scattering data. This method integrates reciprocal space WAXTS simulations, computed from MD atomistic models of bimetallic NPs, with a supervised deep learning (DL) approach based on a convolutional neural network (CNN) regressor.

To demonstrate its effectiveness, we apply this methodology to the analysis of WAXTS pattern extracted from Grazing Incidence Wide-Angle X-ray Scattering (GIWAXS) data, collected *in situ* during the growth of Ag–Co NPs formed by vapor deposition of Co atoms onto predeposited Ag seeds under UHV (Ultra High Vacuum) conditions at room temperature. The Co–Ag system is particularly interesting because it exhibits weak miscibility, a large lattice mismatch and a high cohesive energy difference, favoring a strong segregation tendency, especially in NPs. However, the configuration of Co deposition on an Ag seed is strongly metastable, contrasting the influence of thermodynamic with the growth kinetic conditions.

The input data contain limited structural information, both considering the narrow available Q range (2.3–6.8 Å^–1^, Q being the momentum transfer of the elastically scattered X-rays) and low signal-to-noise ratio (SNR), constraints that are typical of time-resolved experiments, even when performed at synchrotron facilities. Despite the particularly challenging task, the model predictions prove to be highly robust in tracking the out-of-equilibrium growth process of Ag–Co NPs. The resulting picture is consistent with existing theoretical reconstructions and, overall, goes beyond previous interpretations that relied on multiple complementary techniques.^45^ These results highlight the strong potential of the developed method to predict structural and microstructural features of alloy NPs while overcoming the time-consuming and highly manual data modeling procedures that have traditionally limited WAXTS data analysis, even when applied to nonideal experimental datasets.

## Results and Discussion

### Deep Learning Workflow and Data Curation

In this work, similar to other integrated WAXTS-DL applications,^80–83^ each X-ray scattering dataset is treated as a holistic profile, analogous to an image. To train and test our model, a library of WAXTS pattern simulations is first generated, computed by the DSE using a bottom-up strategy, as outlined in Figure 1a. The atomistic models employed here for DSE calculations cannot be reliably derived from geometric or crystallographic considerations alone owing to the unique structural variability of bimetallic NPs and the kinetic control governing their growth process. To capture not only the equilibrium structures but also metastable Ag–Co configurations, particularly relevant during the early stages of growth, as well as their evolution pathways, MD calculations are employed to build the starting atomistic models (*Step 1: MD calculations* in Figure 1a).^50^ This computational approach, capable of handling a relatively large number of atoms at finite temperature while keeping computational costs acceptable (unlike, e.g., Density Functional Theory), enabled the construction of an atomistic database of 140725 Ag–Co clusters of (randomly selected) number of atoms between 2 and 1500 (up to ∼ 3.4 nm), created at 5000 K and subsequently equilibrated at room temperature for 0.2 ns. As a result, the database intentionally samples NP configurations exhibiting a significant degree of structural disorder (although a fraction of relatively crystalline particles can also be found) yielding structures that are more representative of the out-of-equilibrium, kinetically trapped NPs observed during the *in situ* experiments than of idealized Mackay or anti-Mackay icosahedra and chiral-shell structural motifs. Using the Cartesian coordinates extracted from MD, interatomic distances and the corresponding DSE-WAXTS simulations are computed. (*Step 2. DSE simulations* in Figure 1a). At this stage, a set of 140725 DSE-WAXTS patterns is generated using reference conditions that are rather standard for fast (*e.g. in situ*, *operando*) synchrotron data acquisition (5.0° – 55° *2θ* range, step 0.01°, wavelength = 0.56 Å). The DSE-WAXTS simulations required ∼ 3 h on a standard personal computer. Details of the MD calculations and DSE simulations are provided in the Methods section. To bridge the gap between calculated and experimental patterns, we apply a physics-informed data augmentation (*Step 3. Data augmentation and standardization* in Figure 1a) according to which each of the 140725 DSE patterns is combined with noise levels randomly selected within 15 ≤ *SNR* ≤ 115, following a Poisson distribution.^84^ A visual representation of DSE simulations with extreme SNR values is provided in Figure 1b. A data standardization is then applied to all input datasets (including DSE simulated patterns and analyzed experimental data), and involving the following operations: *i)* the *x-*axis of the X-ray patterns is converted from *2θ* to *Q*, being *Q* = 4*π*sin *θ*/λ independent from the wavelength *λ* used for the experiment or pattern calculation. This is particularly convenient for synchrotron data, which may be collected using various X-ray photon energies. *ii)* A suitable *Q-*range (2.0 Å^–1^ ≤ *Q* ≤ 7.5 Å^–1^) is selected from the input data (2.3–6.8 Å^–1^ is the measured range of the GIWAXS data) to remove the need for manual range specification for the input vector to the CNN. If an input pattern exceeds this range, it is automatically adjusted; if shorter, the missing intensities are padded with a constant value equal to the average of the first (for extending *Q*_*min*_) or last (for extending *Q*_*max*_) five intensity points, to mitigate noise-induced fluctuations. This approach ensures compatibility across diverse experimental setups. *iii)* The input data are then resampled using a spline interpolation to yield 1000 equispaced points within the selected *Q*-range (*Q*-step = 0.0055 Å^–1^). *iv)* Finally, each pattern is *normalized* to the same integral area, ensuring comparability among *in silico* signals and those collected under different experimental conditions. The result of this workflow is a 1D equispaced vector (1000 × 1 × 1, intensities only) for each DSE simulation in the database.

**Figure 1:**
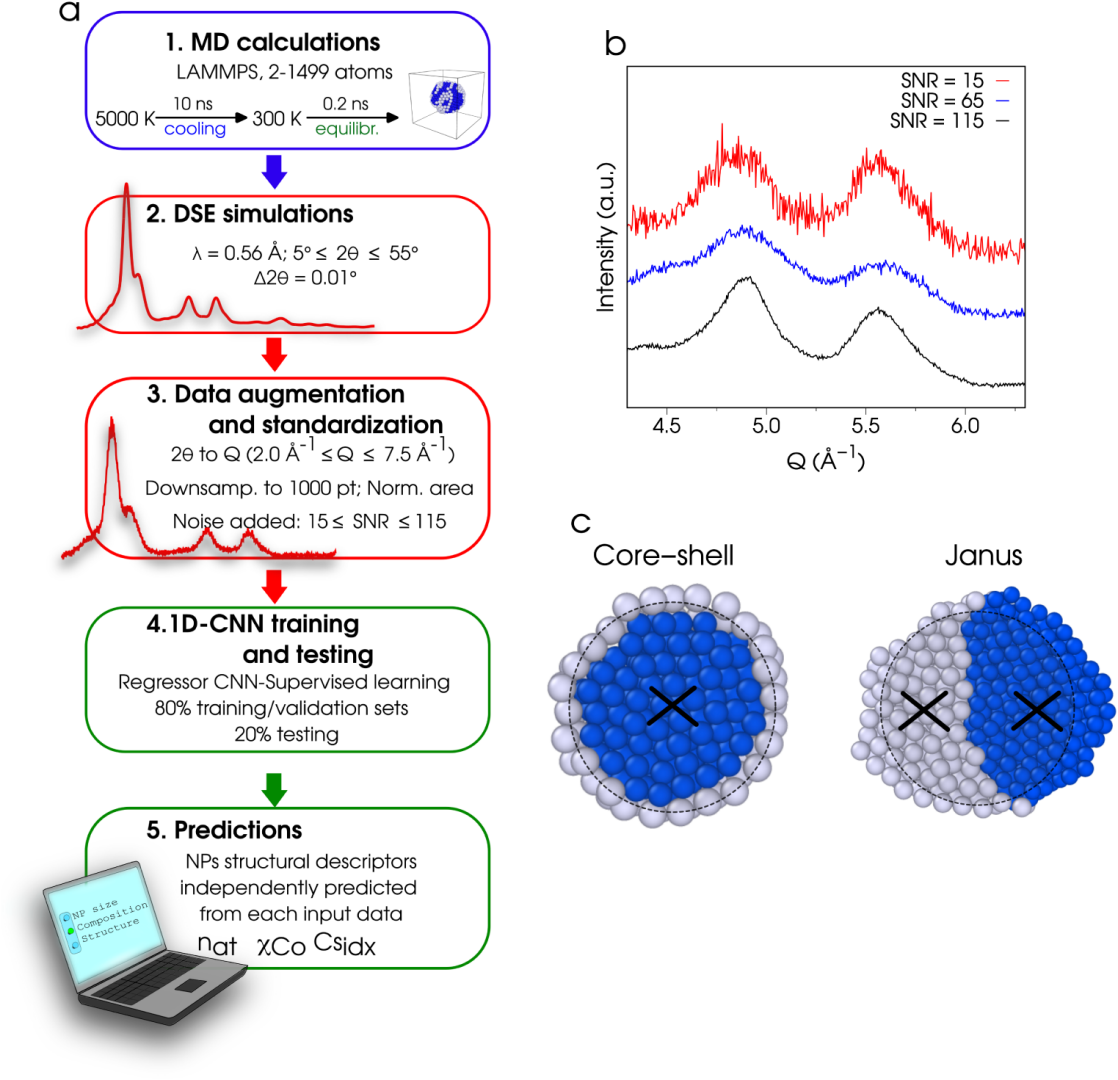
(a) Schematics of the pipeline for the training/testing database generation. It includes MD simulation of the Ag–Co clusters, calculation and standardization of the DSE-WAXTS patterns to feed the CNN regressor providing three structural descriptors (*n_at_*, *χ_Co_*, and *CS_idx_*) as output for each input data. (b) Selected region of the DSE patterns illustrating the variety of SNR used in the training/testing set. From the bottom to top (*y*-offset applied for clarity): highest (*SNR* = 15), medium (*SNR* = 65) and lowest (*SNR* = 115) noise levels. (c) Schematic representation of core–shell and Janus particles, with Ag shown in gray and Co in blue. The dashed circle denotes the sphere having radius of gyration $R_{g}=\sqrt{\frac{3}{5}}R$ of the particle radius, while the black crosses mark the center of mass of each atomic species portion within each cluster type (coincident in the core–shell).

These vectors are assembled into a comprehensive matrix used to train the CNN in a supervised manner on three selected NP descriptors (*Step 4. 1D-CNN training and testing* in Figure 1a). The architecture of the CNN for regression analysis, inspired by ref 85, processes 1D DSE patterns through four convolutional layers followed by a dense block and a three-neurons output layer. Full details on the CNN architecture and results from the hyperparameter exploration used to optimize the model are provided in the Methods and the Supporting Information (Supplementary Text and Figure S1). Finally, the three structural descriptors used for the supervised CNN training/testing are predicted (*Step 5. Predictions* in Figure 1a), allowing the evolution of the Ag–Co NPs during Co deposition to be tracked. These descriptors are itemized and deeply discussed in the next section.

### Growth Parameters and Deep Learning Model Optimization

As reported in the final step of Figure 1a (*Step 5. Predictions*), three parameters are selected to characterize the growth evolution of the Ag–Co NPs: *i)* the clusters size, expressed in terms of number of atoms (*n_at_* = *n_Ag_* + *n_Co_*); *ii)* the clusters composition, expressed as Co fraction, $\chi_{Co}=\frac{n_{Co}}{n_{at}}$ (while *χ_Ag_* = 1 – *χ*_*Co*_); *iii)* an order parameter that distinguishes spatial arrangement and atomic ordering in bimetallic NPs. In this regard, the large difference in cohesive energies (∼34%)^86^ and atomic radii (∼19%)^87^ between Ag and Co (lower cohesive energy and larger radius for Ag) suggests the formation of strongly segregated structures, such as Janus-like or core–shell configurations,^46,47,88^ while occurrence of surface segregation or core–shell ordering depend mostly on the relative abundance of the two metals. When forming clusters, Ag has a lower surface energy than Co,^86^ and it is expected to preferentially segregate to the surface and act as shell element, while Co, characterized by stronger Co–Co interactions, tends to occupy the core.^86^ To describe a continuous transition between Janus-like and core–shell motifs, including intermediate configurations, and following the approach reported in ref 36, we implemented the so-called *Core–Shell index* (*CS*_idx_), schematically illustrated in Figure 1c and detailed in the Methods section. This parameter provides a quantitative measure of spatial separation and compositional ordering within the NPs. It is designed to be negative for Janus-like arrangements and positive for core–shell configurations, increasing as the core–shell character strengthens.

As described above, our database consists of 140725 physically augmented DSE pattern simulations corresponding to an equal number of MD optimized Ag–Co clusters, randomly sampled in size (5 ≤ *n_at_* ≤ 1499) and composition (0.0 ≤ *χ_Co_* ≤ 1.0) and with core–shell index falling in the −0.74 ≤ *CS_idx_* ≤ 2.0 range. This database is divided into training (80%) and testing (20%) subsets for the CNN regressor. Figure S2 shows the mean absolute error (*MAE*) loss function over epochs for both the training and validation (10% of the original training set) sets, demonstrating a stable convergence of the developed architecture.

The performance of the regressor on the test set in predicting the three selected Ag–Co clusters descriptors (*n_at_*, *χ_Co_*, and *CS_idx_*) is evaluated by using the parity plots reported in Figure 2a-c, which compares predicted and true values and enables rapid identification of potential outliers.

**Figure 2:**
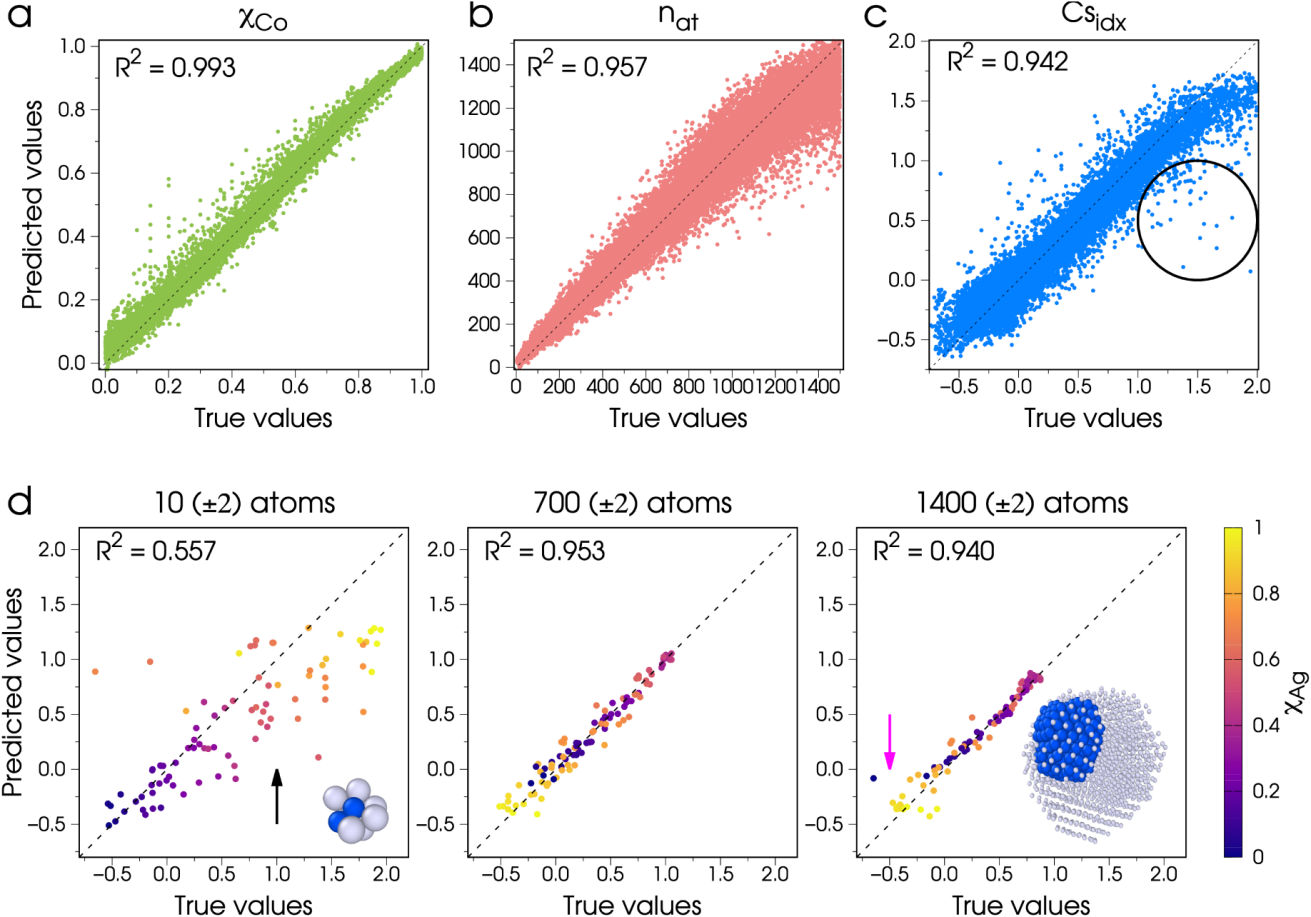
Parity plots of the CNN regressor on the test set for the three target parameters. Predictions *vs* true values are reported for: (a) *χ_Co_* (Co fraction in the NPs); (b) *n_at_* (total number of atoms in the NPs) (c) *CS_idx_* (core–shell index of the NPs). The black circle highlights the outliers discussed in the text. *R* ^2^ is the coefficient of determination. (d) Parity plots for the *CS_idx_* evaluated for clusters with selected *n_at_*, spanning very small (10 atoms, ∼ 0.5 nm), intermediate (700 atoms, ∼ 2.8 nm) and large (1400 atoms, ∼ 3.4 nm) NPs, from left to right. Scatter points are color-coded depending on *χ_Ag_*, according to the color bar on the right. Arrows indicate the outliers discussed in the text, and representative MD cluster models are shown in the insets for visual reference.

The accuracy of each predicted parameter is quantified using the coefficient of determination (*R* ^2^) with values in the 0.942 ≤ *R* ^2^ ≤ 0.993 range confirming a very good overall performance of the regressor. Comparable results are obtained across different kernels tested, as detailed in the Supporting Information. The best agreement is observed in Figure 2a for predictions of *χ_Co_* (*R* ^2^ = 0.993), consistent with the high sensitivity of X-ray scattering patterns to compositional variations of the nanoalloys that modulate the relative intensities of Bragg peaks. A slightly larger deviation is observed in Figure 2b for size predictions (*R* ^2^ = 0.957), particularly for clusters with *n_at_* > 1000 (corresponding to above 3.0 nm Ag–Co clusters). This is consistent with less accurate size determination from WAXTS patterns from defective NPs in the presence of a high concentration of structural defects.^89^ The *CS_idx_* plot in Figure 2c shows the lowest *R* ^2^ of 0.942 and systematic deviations, highlighted with the black circle, are observed mostly at high values. To clarify the origin of these discrepancies, parity plots are grouped in Figure 2d by NPs size (±2 atoms). The deviations that are observed at high *CS_idx_* values (1.0 < *CS_idx_* < 2.0) correspond to very small clusters (∼10 atoms, ∼ 0.5 nm, Figure 2d left), indicating that, in this ultrasmall size regime, *CS_idx_* carries little physical meaning, explaining the reduced prediction accuracy. These deviations rapidly diminish as the cluster size increases, and a very good correlation is already archieved upon reaching approximately 80 atoms (∼1.2 nm), as shown in Figure S3. Small deviations appear at very low *CS_idx_* (∼−0.5), primarily involving larger clusters (∼1400 atoms, Figure 2d right) exhibiting Ag-rich composition (*χ_Ag_* > 0.85). In these cases, the discrepancy likely arises from subtle variations in the degree of Ag coverage around a slightly off-centered Co core.

### Tracking Ag–Co NPs Growth from *in situ* GIWAXS Data

Following validation, the developed CNN regressor is applied to extract atomic-precise parameters and to monitor the growth of Ag–Co NPs from synchrotron *in situ* GIWAXS measurements.

During the experiment, bimetallic NPs are grown at room temperature starting from an Ag equivalent thickness of 1.1 monolayers (MLs, 1 ML = 1.5 × 10^15^ at cm^–2^), anchored on an amorphous carbon substrate. Up to 5.4 MLs of Co are subsequently deposited by vapor deposition under UHV conditions, as described in the Methods section. GIWAXS patterns are collected at 16 keV every ∼ 14 min in the range 2.3 Å^–1^ ≤ *Q* ≤ 6.8 Å^–1^ for a total of 7 h.^45^ GIWAXS data of the initial Ag seeds are well fitted by magic-number icosahedral clusters of different sizes, leading to NPs of approximately 1.7 nm on average, and size dispersion σ = 0.3 nm (details in the Supporting Information and in Figure S4). In the following, for these Ag seeds we refer to the size extracted by the DSE data analysis. Indeed, the CNN-predicted size is expected to be less robust, since the MD database was built for bimetallic clusters. As highlighted in Figure 3a, the compositional evolution of the Ag–Co NPs can be qualitatively followed by monitoring the intensity ratio of the first Ag and Co peaks, at *Q* = 2.73 Å^–1^ and *Q* = 3.11 Å^–1^, respectively; the Co peak progressively increases in intensity at the expense of the Ag peak as Co is deposited. In their seminal study, Andreazza *et al.* ^45^ proposed a tentative interpretation of the growth mechanism of these bimetallic NPs by combining GISAXS (grazing incidence small angle) data modeling, GIWAXS (including anomalous scattering) measurements, and MD simulations. The latter suggested that the Ag–Co clusters adopted icosahedral structures (similar to the Ag seeds) but retained a high degree of structural disorder because equilibrium structures are never reached under the *in situ* growth conditions. This premise underscores the intrinsic complexity of atomistic models for bimetallic NPs. When combined with the limited information encoded in the GIWAXS patterns (restricted Q-range and high noise level, Figure 3a), it makes conventional data fitting approaches rather unfeasible, as in this case. These limitations motivated the development and application of our DL framework, leveraging the AI potential to undertake the challenging task of extracting quantitative information on the NPs structural configuration (*CS_idx_*), growth (*n_at_*) and composition (*χ_Co_*) directly from the *in situ* GIWAXS data. An overview of the three predicted descriptors obtained with the optimized CNN architecture is provided in Figure 3b-d. For the discussion, only a representative subset of predictions along the growth evolution is displayed. Predictions for the complete set of data, and for slightly different CNN architectures explored, are available in Figure S5; the corresponding numerical values are reported in Table S1.

**Figure 3:**
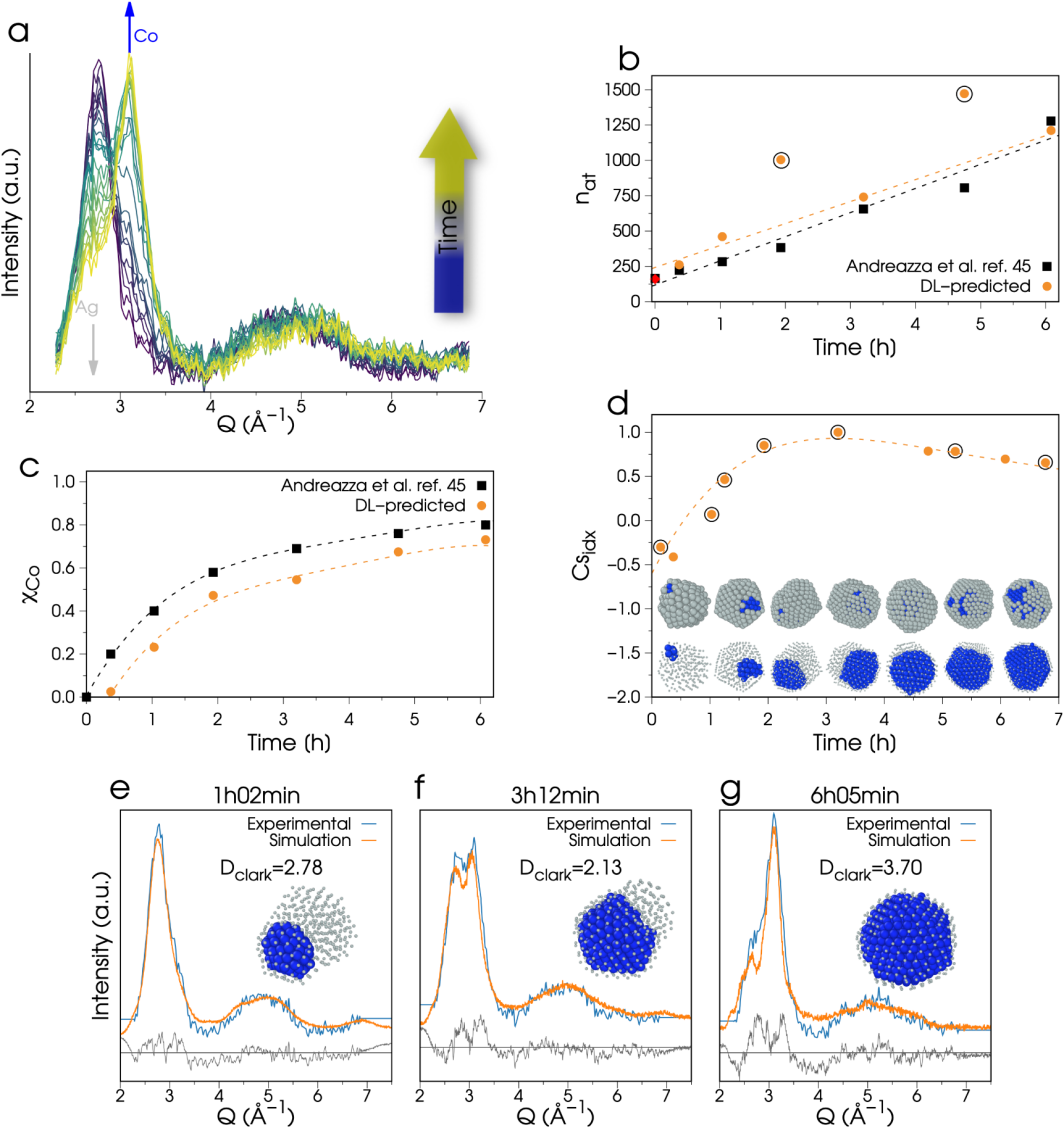
(a) *In situ* GIWAXS data collected during ∼ 7 h of Co vapor deposition on predeposited Ag seeds (∼1.7 nm), with deposition time progressing from blue to yellow shading. The evolution of the first diffraction peaks of Ag and Co, located at Q = 2.73 Å^–1^ and Q = 3.11 Å^–1^, respectively, is highlighted with gray and blue arrows. In panels b and c, predictions *vs* deposition time from the CNN regressor (orange line) are compared with values from ref. 45 extracted from GISAXS measurements (black trace): (b) total number of atoms in the Ag–Co clusters, *n_at_*, for selected growth stages. The red diamond point refers to the size of predeposited Ag seeds extracted from the DSE fit to the experimental data, in line with the GISAXS size; (c) NPs composition expressed as Co fraction *χ*_*Co*_; (d) *CS_idx_* parameter quantifying the structural configuration of the NPs. Circled points correspond to the MD atomistic models shown in the inset. Co atoms are in blue, Ag atoms in gray. Ag atoms are rendered with a reduced diameter to better visualize the Co distribution inside the cluster. The *CS_idx_* evolution reveals the transition from Co atoms initially decorating the NP surface in a Janus-like configuration to their progressive infiltration into the NP core and the emergence of core–shell structures. The dashed lines in panels (b-d) are provided as visual guides. Panels (e-g) show the best-match between input GIWAXS data (blue traces, including padded regions) and DSE simulations (orange traces) indentified by the search-match procedure. These results are selected after 1h 2 min of Co deposition (e, early Ag-rich stage), 3h 12 min (f, intermediate stage with comparable Ag and Co contents) and 6h 5 min (g, final Co-rich stage). The corresponding *D_Clark_* values (calculated excluding the padded regions) are reported.

Figure 3b shows the evolution of the predicted Ag–Co NPs size, expressed as *n_at_* (orange trace). For comparison, the GISAXS-derived sizes reported in ref. 45 are also shown (black trace), after converting the NP volumes to *n_at_* (see the Supplementary Text and Table S2 in the Supporting Information for details). The overall size evolution is appropriately grasped by the CNN predictions and exhibits a reasonable trend consistent with the independent GISAXS determination, although the predicted NP sizes are occasionally overestimated relative to the GISAXS-based values. This behavior suggests that size estimation from the GIWAXS patterns remains a weak point, which is not unexpected, given the known difficulty in disentangling size- and defects-induced peak broadening for small, defective NPs using WAXTS data alone.^89^

The predicted NPs composition (*χ_Co_*) shown in Figure 3c (orange trace), deviates from the nominal Co-deposited quantity (black line). It is systematically lower by approximately 12% on average compared to the nominal values reported in ref 45, and about twice the reported experimental uncertainty of 5%. This discrepancy is consistent across the different CNN architectures tested (Figure S5) and becomes particularly meaningful when considering that the predicted values correspond to the actual NPs composition, whereas the nominal ones reflect the total amount of Co deposited as monolayers. This finding therefore indicates that not all the incoming Co atoms are incorporated into the NPs and that a small portion of Co remains on the substrate, most likely in the form of isolated atoms or very small clusters bound to the amorphous carbon surface. This interpretation aligns with previous computational work indicating that Co atoms exhibit very low mobility on the amorphous carbon substrate, which limits their full incorporation into the Ag–Co NPs.^44^

The structural evolution of the NPs resulting from the predicted *CS_idx_* (not available in ref. 45) displayed in Figure 3d is particularly interesting. At the early growth stages, within the first hour, this parameter ranges from – 0.3 to 0.3, indicating a markedly off-center, quasi-Janus configuration. As Co deposition progresses, *CS_idx_* increases toward values of 0.8–1.0 over approximately 3 h, corresponding to a more symmetric core–shell-like arrangement.

Throughout this continuous evolution from Janus-like to core–shell, the Co atoms, initially attached asymmetrically at the NPs surface, progressively infiltrate into the clusters core (as highlighted by the atomistic models in the inset of Figure 3d), consistent with their bonding features. The most core–shell-like configuration (*CS_idx_* = 1.0) is reached at *χ_Co_* = 0.55 (∼3 h, Figure 3c), in line with MD simulations predicting core–shell structures when Co concentration exceeds 40–45%.^86^ As the Co content continues to increase, the available Ag atoms become insufficient to fully cover the growing Co-rich core, causing *CS_idx_* to decrease down to ∼ 0.7 and indicating a further evolution toward quasi-Janus configurations.

These observed trends remain essentially unchanged when varying the random seed used to select the DSE simulations assigned to the training (80%) and testing (20%) sets of the CNN, as shown in Figure S6.

### Validation of the CNN Predictions by Measuring the Best Match with GIWAXS Data

To assess the robustness of the developed model in retrieving the evolution of bimetallic NPs during Co deposition on Ag seeds, we implemented a search-match algorithm aiming at identifying, within the training/testing database, the DSE pattern of the cluster(s) most representative of the CNN-predicted descriptors (that is, clusters with the closest values to *n_at_*, *χ_Co_* and *CS_idx_*) to be compared to the GIWAXS data. The similarity between the selected DSE patterns and GIWAXS data is quantified using the Clark distance (*D_Clark_*) agreement index.^90^ Details of the procedure are given in the Supporting Information and a schematic workflow is shown in Figure S7. The CNN-predicted structural descriptors and those of the corresponding DSE patterns are reported in Table S3 for the entire GIWAXS sequence. A graphical example of the agreement between the best candidate DSE simulations and the experimental data is shown in Figure 3e-g for selected growth times, along with the related *D_Clark_* values. For comparison, we applied a similar matching procedure to the DSE simulations most representative of the (experimentally derived) NP size and composition reported in Andreazza *et al.* ^45^ (Figure S8). The *D_Clark_* indices remain systematically higher than the previous case throughout the entire growth process, further supporting a closer correspondence of the CNN-predicted NPs features to the GIWAXS data.

The CNN model performs particularly well during the early and intermediate growth stages, where the inversion of the main Ag and Co diffraction peaks occurs (Figure 3f), corresponding to a time window that is particularly relevant for assessing the compositional evolution of the clusters. At longer times (>4 h, Figure 3g), the increased cluster size leads to somewhat larger prediction errors, consistent with the model performance on the testing set (Figure 2a).

Overall, the very good agreement between the experimental GIWAXS patterns and the DSE simulations computed from the CNN-predicted parameters supports the growth mechanism of bimetallic NPs obtained through our DL approach. Worth noting, this approach uniquely provides the evolution of Ag–Co NP stoichiometry, revealing slight but systematic deviations from the mechanism inferred solely from the nominal Co deposition quantities reported in ref. 45.

### Retrieval of Ag–Co NPs Growth Evolution from Larger Ag Seeds

Following the initial validations of the developed CNN regressor on bimetallic NPs for which independent size and composition estimations were available, the same model is applied to a second set of *in situ* GIWAXS measurements. These synchrotron data were collected under conditions different from the previous experiment, that is using a larger amount of initial Ag atoms (2.4 MLs, corresponding to ∼ 2.8 nm clusters) over a total duration of 6 h, resulting in 4.8 Co MLs (Figure 4a). The DSE analysis of GIWAXS data on the predeposited Ag seeds, using magic-number clusters as in the previous case (Figure S9), indicates a majority population of icosahedral clusters (85.5 w/w %) with a minor fraction of decahedral ones (14.5 w/w%) and yields an initial average size of 2.9 nm with σ = 0.5 nm (842 atoms, σ = 433 atoms) for the icosahedra, which is consistent with the CNN-predicted *n_at_* of NPs at subsequent growth times. This fraction of icosahedral *vs* decahedral structure is in agreement with the reported result (90%) from ref 91 in the same size range of Ag NPs. The CNN-predicted *n_at_* values are reported in Figure 4b (magenta symbols) and show limited changes in NPs size, reaching a maximum of *n_at_* = 1935 (∼3.7 nm) after 6 h of Co deposition. This finding is in agreement with the reference results from the previous Ag–Co series (Figure 4b, orange symbols) and is fully consistent with the modified growth conditions. The CNN-predicted values of the other two structural descriptors are shown in Figure 4c-d (numerical values of the three parameters are summarized in Table S4). The compositional evolution (Figure 4c), characterized by systematically lower *χ_Co_* values than in the previous experiment, is consistent with the use of larger Ag seeds and the shorter overall NPs growth time at nearly comparable deposition rate (0.80 ML/h *vs* 0.77 ML/h). These conditions lead to a reasonably delayed increase of the predicted *CS_idx_* (Figure 4d); this descriptor never reaches the plateau observed in the previous experiment, theoretically awaited at *χ_Co_* ≈ 0.5, a value only barely reached at the end of this new experiment (Figure 4c). As a result, the NPs in this new series are predicted to predominantly adopt quasi-Janus or Janus-like configurations except at the very latest growth stages. As previously observed, the predicted *χ_Co_* values (corresponding to the Co amount effectively incorporated into Ag–Co NPs) remain consistently lower than those estimated from the nominal deposition rate, considering the 4.8 Co MLs deposited onto 2.4 MLs of Ag (nominal *χ_Co_* ∼0.67 *vs* 0.48 predicted).

**Figure 4:**
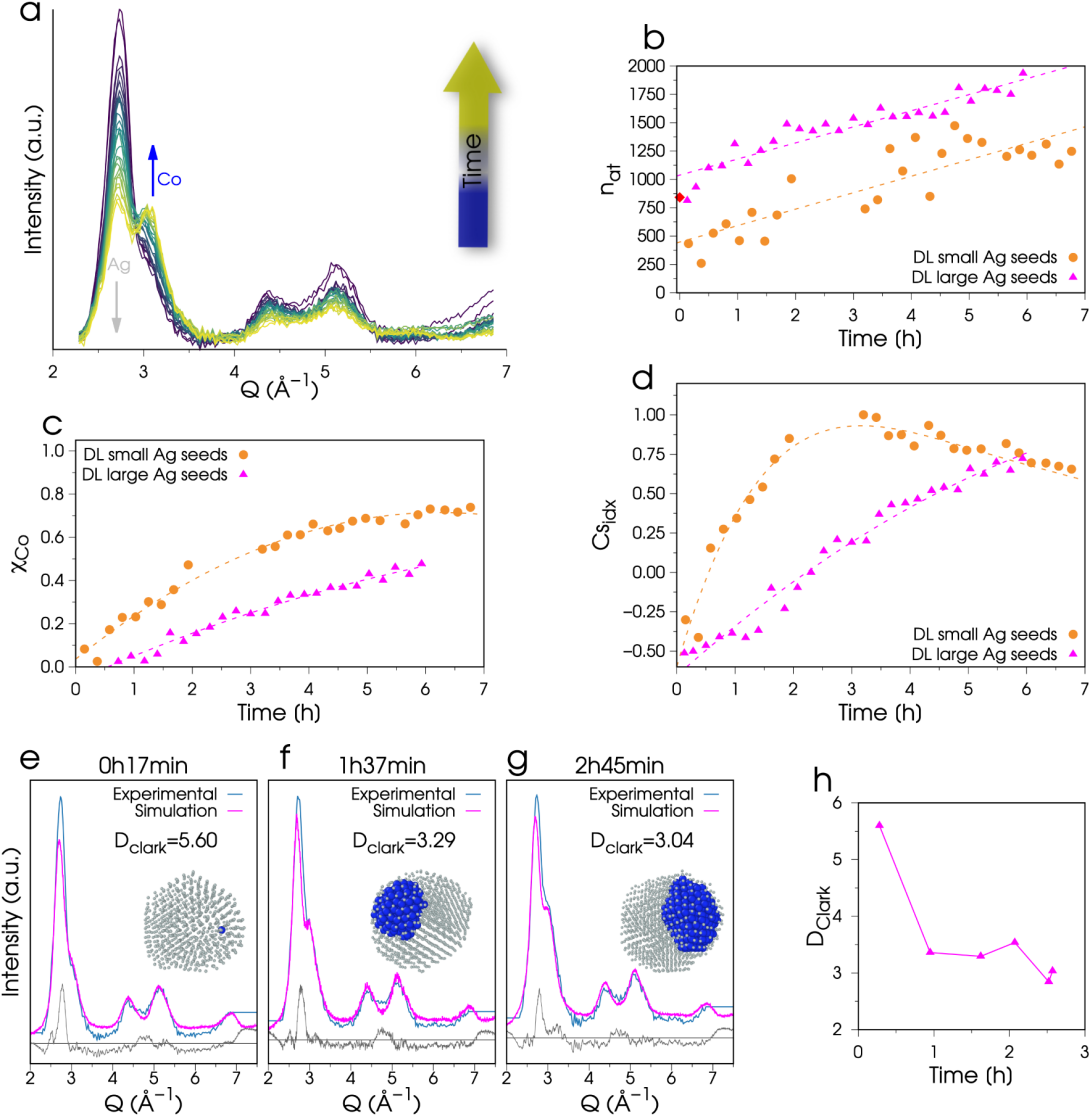
*In situ* GIWAXS patterns collected during Co deposition on large Ag seeds (2.9 nm). The progress of the Co deposition over 6 h is reflected in the modifications of relative intensities of the first Ag and Co diffraction peaks (indicated with gray and blue arrows, respectively). Predictions from the CNN-regressor *vs* deposition time for (b) *n_at_*, (c) *χ_Co_* and (d) *CS_idx_* (magenta lines) are compared with the values predicted on smaller Ag seeds presented in Figure 3 (orange trace). The red diamond point in b) refers to the size of predeposited Ag seeds extracted from the DSE fit to the experimental data The dashed lines in panels (b-d) are provided as visual guides. Panels (e-g) show GIWAXS data (blue traces) and best DSE simulations identified by the search-match procedure (magenta traces), for selected growth stages: (e) early Ag-rich stage at 17 min and two intermediate stages at (f) 1h 37 min and (g) at 2h 45 min. For each stage, the corresponding *D_Clark_* and a MD atomistic model are shown. (h) *D_Clark_* *vs* growth time for the selected growth stages.

Once again, the reliability of the CNN predictions are evaluated via the search-match algorithm described in the previous section. The matches with the GIWAXS data are shown in Figure 4e-g at selected stages, and the corresponding numerical predictions are listed in Table S5. The selected times are limited to the first 3 h of growth, due to the upper limit of the available clusters size in the training database (1499 atoms, ∼ 3.4 nm); on the other hand, the CNN regressor can extrapolate beyond this limit and provides size predictions up to 1935 atoms (∼3.7 nm) after 6 h of experiments.

As indicated by the *D_Clark_* values in Figure 4h, the agreement between experimental and predicted patterns improves over time. The *D_Clark_* values (>5) at the earliest growth stage can be attributed to preferred orientation effects (Figure 4e), commonly observed for faceted NPs deposited on flat substrates.^80^ It is worth noting that a slightly different CNN model, tested for comparison, predicts a somewhat larger initial Ag–Co cluster size of 1250 atoms (∼3.4 nm), followed by only modest size increase up to 1650 atoms (∼3.5 nm) by the end of deposition (Figure S10). Despite the competitive *D_Clark_* values, particularly at the earliest stages (<2h), this alternative model yields initial Ag–Co clusters sizes that are too large when compared with the size of predeposited Ag-seeds (2.9 nm, 842 atoms, Figure S9).

## Conclusion

In this work, we have developed a Deep Learning model that enables accurate and fully automated quantitative analysis of Ag–Co bimetallic nanoalloys, in terms of microscopic descriptors. The analysis tracks the NPs growth evolution, monitored *in situ* by real time GIWAXS experiments, using X-ray scattering datasets as the sole input information for predictions. The DL model is trained on *in silico*-generated WAXTS patterns calculated from MD snapshots of Ag–Co NPs. This approach paves the way for the progress of AI-based strategies for characterizing NPs under out-of-equilibrium conditions.

To capture the metastable configurations that are not achievable within conventional crystallographic frameworks, a large library of MD generated atomistic models of bimetallic NPs is built, spanning a large range of sizes, compositions, and structural motifs retaining a high degree of defects. From these clusters, DSE-simulated WAXTS patterns are generated covering a wide parameter space used to train a CNN-regressor. The model was optimized through extensive hyperparameter exploration to achieve a balance between predictive accuracy and generalization.

When applied to *in situ* GIWAXS datasets collected during Co deposition on Ag seeds, the model successfully reconstructs the full Janus-to-core–shell evolution pathway and its correlation with the compositional evolution of the NPs, in fair agreement with existing computational studies.^45,86^ The application of this model also clarifies that not all deposited Co atoms are incorporated into the NPs, owing to their limited mobility on the amorphous carbon substrate, an effect that influences both the final composition and structure of the particles under kinetically controlled growth. We highlight that the AI analysis from a pertinent WAXTS pattern library is capable of extracting the NPs composition information directly from the measured GIWAXS patterns (a quantity otherwise inaccessible in such type of experiments), and which further validates the predicted low mobility of Co on the substrate. This finding points to the key role of kinetic control in Ag–Co NP growth, governed by a diffusion-limited regime that alters the final NPs composition compared to previous interpretations. Figure 5 summarizes the main findings of this work in comparison with what was previously known from the literature.

**Figure 5:**
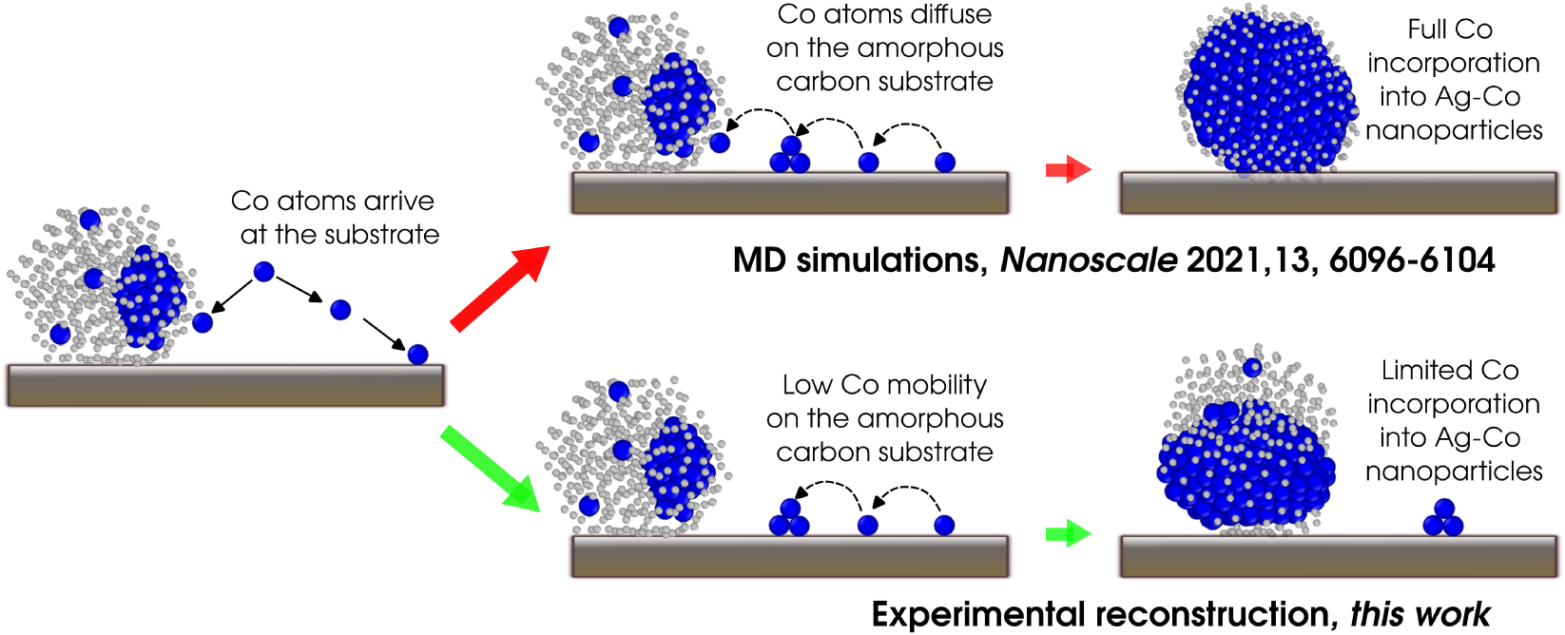
Schematic overview of the main findings obtained from the developed MD-TS-DL framework, illustrating the reconstruction of the formation and growth mechanisms of Ag–Co NPs from *in situ* GIWAXS datasets collected under out-of-equilibrium conditions. Following the green arrows, the elucidation of the Janus-to-core–shell evolution pathway, and the kinetic control determined by the limited mobility of Co atoms on the amorphous carbon substrate, which results in NP compositions that differ from the nominal values. A comparison with what was previously known from the literature^45^ is also shown (red arrows).

The successful extension of the developed CNN regressor to an additional *in situ* GIWAXS experiment, involving larger Ag seeds, further demonstrates the predictive capabilities and transferability of this approach. This conclusion is strengthened by the striking similarity between the simulated DSE patterns derived from the CNN-predicted NP descriptors and the GIWAXS data across both experiments.

Altogether, these results confirm that the developed Deep Learning methodology provides consistent and physically meaningful insights, demonstrating its potential as a standalone tool for extracting valuable structural and size descriptors from X-ray scattering data from complex bimetallic NP systems. Its ability to deliver rapid and automated information makes it particularly well-suited for *in situ* and *operando* experiments where fast response is essential. By providing atomic-level insights across the entire NPs growth process, this combined MD-DSE-DL approach has the potential to significantly accelerate the development and optimization of functional nanomaterials, offering capabilities and time scale responses that are difficult to achieve through conventional, time-consuming fitting procedures.

## Methods

### Molecular Dynamics (MD) Simulations

The MD calculations were performed to generate a large-scale database of Ag–Co atomistic clusters, enabling the capture of both equilibrium and metastable NPs configurations. All calculations were carried out using the LAMMPS software package, using a simulated annealing protocol employing the Velocity Verlet integration algorithm,^92^ a Nosé-Hoover thermostat,^93,94^ and semiempirical tight-binding second moment approximation potentials.^95,96^ This method has been already successfully applied to a variety of nanoalloys systems.^45,97^

The MD calculations workflow for all clusters of the database can be summarized as follows. First, the total number of atoms was randomly selected from a uniform distribution between 2 and 1500 atoms. Next the number of Ag atoms was randomly chosen between 1 and *n_at_* – 1 (where *n_at_* is the total number of atoms), with the remaining atoms assigned to Co. Ag and Co atoms were then randomly positioned inside a box whose edge length was determined from the experimental bulk densities of the two species and their respective concentrations. During atom placement, a minimum interatomic distance constraint was enforced to avoid unphysically high-energy configurations. This initial randomness ensures structural diversity and prevents bias toward specific structural motifs.

Simulated annealing was used to induce NPs formation. The initially disordered, gas-phase configuration was cooled from 5000 to 300 K over 10 ns using 5-million-time steps of 2 fs each. This time scale and cooling rate were selected to balance adequate exploration of the potential-energy landscape with computational efficiency. After annealing, each system was equilibrated at 300 K for an additional 0.2 ns. The final configuration of each cluster was saved as an.*xyz* file containing the 3D atomic Cartesian coordinates, accompanied by the cluster structural descriptors (*n_at_*, *χ_C__o_*, and *CS*_*idx*_) used as labels for subsequent CNN training. The resulting MD database contains 140725 entries spanning a broad distribution of NP sizes and structural motifs. The Cartesian coordinates of these clusters, stored as *xyz* files, were then used as input for the corresponding DSE X-ray pattern simulations.

### Core–Shell Index (CS_idx_) Definition

To discriminate between core–shell, Janus and any intermediate configurations (such as quasi-Janus), the monotonic parameter *CS_idx_* is defined following the approach presented in ref 36. This parameter is designed to be small for Janus-like configurations and large for core–shell ones:

$$CS_{idx}=2\left|\frac{n_{Ag}^{out}}{n_{Ag}^{out}+n_{Co}^{out}}-\frac{n_{Ag}}{n_{tot}}\right|+2\left|\frac{n_{Ag}^{in}}{n_{Ag}^{in}+n_{Co}^{in}}-\frac{n_{Ag}}{n_{tot}}\right|-\frac{d_{com}}{2r_{g}}$$

where $n_{Ag}^{out}$ and $n_{Co}^{out}$ are the numbers of Ag and Co atoms in the outer shell of the particle, defined as the atoms located at distances larger than 80% of the particle radius of gyration (i.e., *R_g_*, the root-mean- square distance of atoms from ther center of mass). Similarly, $n_{Ag}^{in}$ and $n_{Co}^{in}$ represent the numbers of Ag and Co atoms in the core, corresponding to a distance smaller than 80% of *R_g_*. The first term in the equation compares the Ag fraction in the shell to the overall Ag concentration. These two concentrations are expected to be similar for Janus particles. For core–shell particles, the local and global compositions differ markedly, producing a positive contribution. The second term performs a similar comparison for the particle core.

The third term involves *d_com_*, which is the distance between the centers of mass of Ag and Co atoms:

$$d_{com}=\left|\frac{1}{N_{Ag}}\underset{i\in Ag}{\sum }r_{i}-\frac{1}{N_{Co}}\underset{i\in Co}{\sum }r_{i}\right|$$

This term is expected to be small for core–shell structures (zero in the ideal case, where the Ag and Co centers of mass coincide) and large for Janus configurations, thus providing a negative contribution upon normalization by 2*R_g_*. The prefactors (2 and 1/2) in the formula are chosen empirically to balance the contributions of the different terms. Absolute values were included in the concentration-comparison terms to ensure sensitivity to both Ag-core/Co-shell and Co-core/Ag-shell arrangements.

### Debye Scattering Equation (DSE) Simulations from Atomistic Models

The DSE calculates the average differential elastic cross-section (or the total scattering pattern) of a randomly oriented powder of NPs using the distribution of interatomic distances between atomic pairs, without any assumption of periodicity and order.^54,98^ The modified DSE, implemented in the Debussy Suite,^61,62^ is reported:

$$I\left(Q\right)=\overset{N}{\underset{j=1}{\sum }}f_{j}^{2}\left(Q\right)o_{j}+2\overset{N}{\underset{j>i}{\sum }}f_{j}\left(Q\right)f_{i}\left(Q\right)T_{j}\left(Q\right)T_{i}\left(Q\right)o_{j}o_{i}\frac{\sin\left(Qd_{ij}\right)}{\left(Qd_{ij}\right)}$$

where *Q* = 4πsinθ/λ is the magnitude of the scattering vector (or momentum transfer), λ is the radiation wavelength, *N* is the total number of atom, *f_j_* (*Q*) is the atomic form factor of element *j, d*_*ij*_ is the interatomic distance between atoms *i* and *j*, $T_{j}\left(Q\right)=\left[\exp\left(-\frac{1}{2}\left\langle u^{2}\right\rangle Q^{2}\right)\right]$ is the Debye-Waller factor (in the harmonic approximation, encoding thermal motion and static disorder) where ⟨*u* ^2^⟩ is the isotropic mean-square atomic displacement; *o_j_* is the site occupancy factor associated with each atomic species. The first summation in the above equation includes the contributions of zero distances between one atom and itself and the second term (the interference term) the nonzero interatomic distances *d*_*ij*_ = |*r*_*i*_ - *r*_*j*_|. Starting from the MD atomistic models, the DSE patterns are calculated using the *Debussy* program suite.^61^ Gaussian sampled interatomic distances^99^ and related pseudomultiplicities (speeding up calculation time) are computed directly from the atomic coordinates stored in each *xyz* file for each cluster and encoded into the appropriate database of NPs sampled distances. This database is then used to calculate the DSE pattern for each Ag–Co cluster, by fixing *o_j_* = 1 and ⟨*u* ^2^⟩ = 0.01 Å^2^.^100^ DSE-WAXTS patterns are generated by considering instrumental setups representative for fast (*e.g.* *in situ*, *operando*) synchrotron experiments, using a 5.0° – 55° *2θ* range, a step of 0.01°, and an X-ray wavelength of 0.56 Å. This procedure requires about 3 h on a standard personal computer.

### Convolutional Neural Network (CNN) Architecture

In this work, a one-dimensional CNN inspired by ref 85, is implemented as a supervised DL model to regress three selected structural descriptors (*n_at_*, *χ_Co_*, and *CS_idx_*) from a database of 140725 entries. The databases consists of equispaced WAXTS intensity profiles encoding the scattering features of corresponding MD snapshots of bimetallic Ag–Co atomistic models. Details of the network architecture are provided in the Supporting Information (Supplementary Text and Figure S1). Training employs the Mean Absolute Error (MAE) as loss function, the Adam^101^ optimizer, and a combination of *ReLU* (hidden layers) and linear (output layer) activation functions. The optimizer learning rate was set to 1 × 10^–4^, controlling the step size of parameter updates at each iteration. To prevent overfitting, early stopping with a patience of 5 epochs was used, monitoring the validation loss (Figure S2). Best-weight restoration was enabled to recover the model weights corresponding to the minimum validation loss. The network is implemented in TensorFlow^102,103^ using Keras (v2.11.0) front-end.^104^

Before training, the three target descriptors are scaled to the interval [0, 1]; predictions are subsequently rescaled to physical units using the inverse transformation. Because neural-network performance strongly depends on both architecture and hyperparameters and no universal design protocol exists, extensive tests were conducted by varying kernel size, activation function, and other structural or optimization-related parameters. Kernel size and activation function were found to be particularly relevant in influencing the CNN outcomes, as detailed in the Supporting Information.

This CNN was trained on 80% of the 140725 DSE simulations, with the remaining 20% of the dataset used for testing. To address potential overfitting issues, 10% of the training set was used as validation set. The evolution of the MAE loss function for both training and validation sets across epochs is reported in Figure S2.

The complete training/validation/testing procedure required approximately 35 min on a GPU-enabled personal computer (Intel Core i7–12700H 20 cores processor; NVIDIA GeForce RTX 3060 graphics card) and about 1h and 20 min on a multicore processor (AMD EPYC 7301 64 cores processor; 188 GB RAM).

### *In situ* Grazing Incidence Wide Angle and Small-Angle X-ray Scattering Experiments

The experimental data used in this work were collected during *in situ* experiments performed at the SIxS beamline of the SOLEIL synchrotron (France).^105^ The experiment was carried out under ultrahigh vacuum (UHV) conditions, enabling simultaneous GIWAXS and GISAXS measurements. Ag–Co NPs were grown *in situ* at room temperature on amorphous carbon substrates by vapor deposition, ensuring high-purity nanoalloys with minimal contamination. Metal evaporation was controlled by applying an electrical tension to the solid metal bars. Deposition rates were previously calibrated by using Rutherford backscattering (RBS), ensuring accurate compositions (within ± 5%) and deposited quantities. During the growth, deposition rates were maintained at 0.5 −1 × 10^15^ atoms cm^–2^ h^–1^ for each metal. The initial pressure was set at 1 × 10^–10^ mbar, and it was increased slightly to 2 × 10^–10^ mbar during the deposition.^45^

A nominal amount of 1.1 MLs (monolayers as equivalent thickness) of Ag was first deposited to form the clusters seed. Following the inverse deposition approach, an additional 5.4 ML of Co was deposited onto. The Co deposition lasted roughly 7 h. The ensemble of GIWAXS patterns relevant for the present work was collected at a photon energy of 16 keV, approximately every 14 min, producing 26 frames covering the full growth process. Measurements were performed at a critical angle α_c_ = 0.11° and an incidence angle α = 0.08°, covering the 2.3 Å^–1^ ≤ Q ≤ 6.8 Å^–1^ range.

For the second GIWAXS dataset discussed in this work, the experiment followed a procedure similar to that described above. In this case, data were acquired approximately every 14 min over a total duration of about 6 h during continuous Co deposition (up to 4.8 ML) onto preexisting larger Ag seeds (2.4 ML), yielding 28 GIWAXS frames covering the full growth sequence.

## Supplementary Material



## Data Availability

All codes developed and implemented in this work can be found in a public repository located at https://github.com/DeByeUSerSYstem/NanoAlloys_WAXS_analyser, https://debyeusersystem.github.io

## Notes

Architecture of the neural network regressor, the approximation used to convert Ag–Co nanoparticle (GISAXS-based) volumes into the corresponding number of atoms, the DSE modeling of GIWAXS data collected on predeposited Ag seeds, and a description of the search-match algorithm developed to identify the DSE simulations representative of the CNN-predicted structural descriptors best matching the GIWAXS data. Supplementary Tables S1–S5 contain additional numerical values of predictions and figures S1–S10 report supplementary graphical outcomes (PDF)
