# Supplementary material for: Tracking Out-Of-Equilibrium Growth of Bimetallic Nanoalloys Using Deep Learning and X‑ray Scattering
Source: ACS Nano. 2026 Jul 21;20(30):21445–58. doi: 10.1021/acsnano.6c09310 (PMC13450433; doi:10.1021/acsnano.6c09310)
Supplement: Supplementary file 1 [file nn6c09310_si_001.pdf]

Supporting Information for

Tracking Out-of-Equilibrium Growth of Bimetallic Nanoalloys Using Deep Learning and X-ray Scattering

Lucia Allara,[1,4] Georg Daniel Förster,[2] Pascal Andreazza,[2] Federica Bertolotti*[1,4,5] and Antonietta Guagliardi*[3,4,5]*

[1] Department of Science and High Technology, University of Insubria, via Valleggio 11, Como 22100, Italy.

[2] Université d'Orléans, CNRS, Interfaces, Confinement, Matériaux et Nanostructures (ICMN), 45071 Orléans, France

[3] Istituto di Cristallografia, Consiglio Nazionale delle Ricerche (*CNR*), via Valleggio 11, Como 22100, Italy.

[4] Total Scattering Laboratory (To.Sca.Lab), Department of Science and High Technology, University of Insubria, via Valleggio 11, Como 22100, Italy.

[5] INSTM, Consorzio Interuniversitario per la Scienza e Tecnologia dei Materiali, Via G. Giusti 9, Firenze 50121, Italy.

1. Convolutional Neural Network (CNN) 3
2. Conversion between Ag-Co nanoparticle volumes and number of atoms 4
3. Identification of DSE simulations from the CNN-predicted structural descriptors via the D_{Clark} indicator 5
4. DSE modeling of the GIWAXS experimental data collected on pre-deposited Ag seeds. 6
5. Supplementary Tables 7
6. Supplementary Figures 11

1. Convolutional Neural Network (CNN)

The architecture of the developed CNN regressor, inspired by ref. 1, is outlined in Figure S1 and consists of the following elements: *i)* an input layer, *i.e.* a *1D input vector* with dimensions 1000×1×1; *ii)* a *1D convolutional layer*, with 32 kernels, and kernel size of 30×1×1 units, moved with a 1×1×1 stride, followed by a *Max Pooling layer* with kernel and stride sizes 2×1×1 producing 32 features to reduce the dimensionality of the system (to 500×1×1). *iii)* A *1D convolutional layer*, with 64 kernels, and kernel size of 25×1×1 units, moved with a 2×1×1 stride (resulting in feature maps of size 250×1×1), followed by a *SpatialDropout layer* (dropout rate = 0.3) for regularization purposes, in which the dropout mask is applied to entire feature maps rather than individual neurons randomly chosen within all feature maps, particularly effective when adjacent neurons are highly correlated (*i.e.* in the early convolution layers). *iv)* a *1D convolutional layer*, with 64 kernels, and kernel size of 20×1×1 units, moved with a 2×1×1 stride, followed by a *Max Pooling layer* with kernel and stride sizes 2×1×1 reducing the feature maps to 63×1×1. *v)* a *1D convolutional layer*, with 64 kernels, and kernel size of 20×1×1 units, moved with a 1×1×1 stride, followed by a *Max Pooling layer* with kernel and stride sizes 2×1×1, producing 64 feature maps of dimensions 32×1×1. *vi)* A dense block composed of: a *flattening step,* aligning all the feature maps in a single 1D vector of size 2048×1; a *fully connected layer* with size equal to 1/3 of the flattened layer (682 neurons); a *Dropout layer* with dropout rate of 0.3 for regularisation purposes; a *fully connected layer* of size 1/4 of the previous layer (170 neurons); a *Dropout layer* with dropout rate of 0.1. *v)* An *output layer* consisting of three fully connected neurons and implementing a *linear activation function* that provides the outputs of the network.

Except for the output layer, a consistent non-linear *ReLU* ($f(x) = \max(0, x)$) activation function was used throughout the network, ensuring efficient and stable training. An alternative sigmoid function ($f(x) = \frac{1}{1+e^{-x}}$) was tested, constraining its outputs in the 0-1 interval. Such a restriction effectively prevents the network from extrapolating beyond the training minimum and maximum values, an ability that is essential for regression tasks. This limitation made the sigmoid-based networks less effective, and this function was finally discarded. “Same” padding with edge-value filling was applied to all convolution and pooling operations.

The neural network was trained on 80% of the 140725 total simulated data, and the remaining 20% of the dataset was used for testing. To address potential overfitting issues, 10% of the training set was used as validation set. The Mean Absolute Error loss function ($MAE = \frac{1}{n}\sum_{i=1}^{n}|y_i - \widehat{y_i}|$, where y_i are the true values, $\widehat{y_i}$ the predicted ones and n the number of observations) was used to optimize and monitor the network across epochs for the training and validation sets (Figure S2). The Adam optimizer with a learning rate 1 10^{-4}, was employed, and an early stopping strategy with patience 5

monitoring the validation loss, and best training weights restoration was implemented. The network was implemented in TensorFlow[2,3] using Keras (v2.11.0) front-end.[4]

The selection of hyperparameters of the deep learning (DL) model, including the number of convolutional layers, the size and number of the kernels, and stride sizes was carried out on a trial-and-error basis. Several plausible versions of the model were tested, and the final architecture was determined based on empirical evaluations, by iteratively testing various hyperparameter choices and evaluating the model performance on previously analyzed experimental data. This iterative process allows for fine-tuning and optimizing the algorithm to achieve the best possible performance and generalization for the specific task. Indeed, there are no strict guidelines or rigorous principles for determining the hyperparameter space in advance.[5]

2. Conversion between Ag-Co nanoparticle volumes and number of atoms

To compare the GISAXS-derived size values reported in ref. 6 with the number of atoms predicted by the DL model, we assumed an icosahedral structure built according to the concentric-shells Mackay model.[7] We used the formula presented in ref. 8 giving the total number of atoms in an *n*-shell icosahedral cluster:

$$N_I(n) = \frac{(10n^3 + 15n^2 + 11n + 3)}{3}$$

and the corresponding cluster equivalent spherical diameter[8]

$$D_I(n) = 1.1964\ (n\ + 0.5)\ a_{fcc}$$

where n is the shell index and a_{fcc} is the lattice parameter of the *fcc* reference structure.

In the present case, calculations are performed by taking the bulk values of a_{fcc} of Ag and Co from ICSD-22434[9] and ICSD-53805,[10] respectively, reduced by 2% to account for the reported lattice contraction of icosahedral structures relative to the *fcc* phase.[8] The final lattice parameters are 3.951 Å for Ag and 3.483 Å for Co.

For deriving $D_I(n)$ of mixed Ag/Co compositions, the a_{fcc_Cl} of the alloyed cluster was estimated as the weighted average of Ag and Co lattice parameters, using the Co fraction (χ_{Co}) as the weighting factor:

$$a_{fcc\_Cl} = \chi_{Co} a_{fcc\_Co} + (1 - \chi_{Co}) a_{fcc\_Ag}$$

The GISAXS analysis in ref. 6, provided the (Gaussian-based) average diameter and height of clusters for each Co deposition stage. From these values the mean nanoparticle (NP) volumes are calculated and converted into an equivalent-sphere diameter (D_{GISAXS}). The two adjacent icosahedral

clusters closest to this size ($D_I(n) \leq D_{GISAXS} \leq D_I(n+1)$) are identified and the weights of their linear combination ($w_n + w_{n+1} = 1$) determined to retrieve the target diameter:

$$w_{n+1} = \frac{D_{eq} - D_I(n)}{D_I(n+1) - D_I(n)}$$

$$w_n = 1 - w_{n+1}$$

Finally, the average number of atoms per sample (N_{at}) was obtained as the weighted average of the corresponding N_I values of these two nearest clusters:

$$N_{at} = w_n N_I(n) + w_{n+1} N_I(n+1)$$

The complete list of samples volumes and compositions (from ref. 6), equivalent-sphere diameters, and the corresponding number of atoms is reported in Table S2.

3. Identification of DSE simulations from the CNN-predicted structural descriptors *via* the D_{Clark} indicator

We developed a strategy to assess the relative reliability of the DL-predicted parameters (n_{at}, χ_{Co}, and CS_{idx}) across the investigated GIWAXS data series. To this end, we implemented a search-match procedure to identify, within the database, the DSE simulation associated with a cluster representative of the predicted parameters (within very small tolerance thresholds) and that most closely matches the experimental GIWAXS pattern at each growth stage. The similarity between the simulated and experimental patterns was quantified using the Clark distance[11] agreement parameter. A schematic flowchart of the procedure is shown in Figure S6.

The method operates in two steps. First, the algorithm searches the database to identify the clusters whose n_{at}, χ_{Co}, and CS_{idx} values are as closest as possible to the target parameters, either those predicted by the CNN or those reported in ref 6. The search thresholds are iteratively slightly relaxed until at least five clusters are found.

In the second step, the algorithm compares the experimental GIWAXS data with the DSE simulation associated with each of the five selected clusters, by calculating the Clark distance (D_{Clark})[11] between each pair:

$$D_{Clark} = \sqrt{\sum_{i=1}^{N} \frac{\left|I_i^{EXP} - I_i^{DSE}\right|}{I_i^{EXP} + I_i^{DSE}}}$$

where I_i^{EXP} and I_i^{DSE} represent the intensities at the ith Q-step of the experimental and simulated patterns, respectively (padded regions are excluded from calculations). Among the five selected DSE patterns, the smallest D_{Clark} value is retained as the final best-matching solution, also supported by an additional visual inspection. This step is consistent with standard practice in powder diffraction,

where the model validation typically combines numerically agreement factors with a visual inspection of the fit quality relative to the experimental data.

Table S3 and Table S5 summarize the outcome of this search-match procedure for the two series of experiments analyzed in the present work. Some graphical results are shown in Figures 3 and 4 of the main text and Figures S7 and S9.

4. DSE modeling of the GIWAXS experimental data collected on pre-deposited Ag seeds.

GIWAXS data collected on pre-deposited Ag seeds were modelled using the DSE approach implemented in the Debussy Program Suite.[12] Populations of icosahedral (I) and decahedral (D) Ag magic-number clusters were generated by adding successive atomic shells around a central atom at the origin (0th shell), up to 20 shells, according to the Mackay (I) and Martin (D) models.[7,13] Atomic coordinates are obtained as described in ref. 8. The n-th shell ($n > 0$) contains $\nu_I(n) = 10n^2 + 2$ atoms for icosahedral clusters, and $\nu_D(n) = 1 + \frac{5n(n+1)}{2}$ atoms for decahedral clusters. Accordingly, the total number of atoms in a cluster with n shells is:

$$N_I(n) = \frac{(10n^3 + 15n^2 + 11n + 3)}{3}$$

$$N_D(n) = \frac{(5n^3 + 15n^2 + 16n + 6)}{6}$$

The generation of Gaussian sampled interatomic distances and the corresponding pseudo-multiplicities are computed directly from the atomic coordinates for each cluster.[14] The Debussy Program Suite is used to calculate the model DSE patterns from Gaussian sampled interatomic distances, encoded into appropriate databases. A log-normal size distribution is assumed, and the model parameters (average size, size dispersion and lattice strain) are optimized against the experimental data.

The data collected on smaller Ag-seeds are well fitted by the icosahedral population of Ag clusters, while for data collected on larger seeds, icosahedral and decahedral populations were included in the model. The best DSE fits are shown in Figures S3 and S8, respectively, along with the refined size distributions. In addition to the average samples sizes, size distributions, and isotropic atomic thermal displacement parameters, a uniform isotropic strain factor was also refined, yielding an approximate ~2% lattice contraction relative to the reference bulk value ($a_{fcc} = 4.032$ Å).[9]

5. Supplementary Tables

Table S1. DL-predicted structural descriptors n_{at}, χ_{Co}, and CS_{idx} from the *in-situ* GIWAXS data, at each Co deposition time. Grey rows indicate the data points used in the comparative analysis shown in Figure 3 of the main text.

Time (h)	n_{at}	χ_{Co}	CS_{idx}
0.15	434	0.08	-0.30
0.37	260	0.03	-0.41
0.58	526	0.17	0.16
0.80	608	0.23	0.28
1.03	460	0.23	0.34
1.25	708	0.30	0.46
1.47	455	0.29	0.54
1.68	685	0.36	0.72
1.93	1005	0.47	0.85
3.20	741	0.55	1.00
3.42	820	0.56	0.98
3.63	1271	0.61	0.87
3.85	1075	0.61	0.87
4.07	1370	0.66	0.80
4.32	851	0.63	0.93
4.53	1230	0.64	0.87
4.75	1473	0.68	0.79
4.97	1361	0.69	0.78
5.22	1327	0.68	0.79
5.65	1204	0.66	0.82
5.87	1262	0.71	0.76
6.08	1212	0.73	0.70
6.33	1311	0.73	0.70
6.55	1136	0.72	0.67
6.77	1247	0.74	0.66

Table S2. For each Co deposition time, GISAXS-derived NP volumes ($\bar{V}_{GISAXS}$) from ref. 6, the corresponding diameters of spheres with equivalent volume (D_{GISAXS}), and the nominal Co fraction (χ_{Co})[6] are listed. The final two columns report the number of atoms $N_I(n)$ (with n shell number of the icosahedral concentric Mackay shell model[7]), in the two adjacent clusters, and the weights w_n from which the corresponding number of atoms (N_{at}) is calculated (as detailed in the Supplementary Text).

Time (h)	V_{GISAXS} (nm^3)	D_{GISAXS}(nm)	χ_{Co}	$w_n, N_I(n), N_I(n+1)$	N_{at}
0.00	2.60	1.71	0	0.89, 147, 309	165
0.37	3.20	1.83	0.20	0.54, 147, 309	222
1.03	3.88	1.95	0.40	0.16, 147, 309	283
1.93	4.93	2.11	0.58	0.71, 309, 561	383
3.20	8.19	2.50	0.69	0.74, 561, 923	656
4.75	9.79	2.65	0.76	0.32, 561, 923	806
6.08	15.40	3.09	0.80	0.28, 923, 1415	1279

Table S3. For each Co deposition time, the structural descriptors n_{at}, χ_{Co}, and CS_{idx} serving as "Target value" input to the search-match procedure described in the Supplementary Text are quoted. DL-predicted values (orange colored rows) and those from ref. 6 (white rows) are compared with the output parameter ranges identified by the search-march procedure and corresponding to the five most similar clusters. The corresponding minimum and maximum D_{Clark} values are reported in the last column. For convenience, sample labels used in Figure 3 of the main text, linked to this analysis, are also given in the first column.

**Time (h) [label]**	**Set**	**Type**	n_{at}	χ_{Co}	CS_{idx}	D_{Clark}
0.37 [22min]	DL predictions	Target value	260	0.03	-0.41	3.52/3.91
		Selected DSE sim.	257/263	0.02/0.03	-0.44/-0.39	
	Ref. 6	Target value	222	0.20	-	4.19/4.38
		Selected DSE sim.	220/223	0.19/0.21	0.31/0.64	
1.03 [1h02min]	DL predictions	Target value	460	0.23	0.34	2.78/2.99
		Selected DSE sim.	458/463	0.23/0.24	0.19/0.47	
	Ref. 6	Target value	283	0.30	-	3.47/3.74
		Selected DSE sim.	281/285	0.39/0.41	1.07/1.46	
1.93 [1h56min]	DL predictions	Target value	1005	0.47	0.85	1.75/1.84
		Selected DSE sim.	1004/1008	0.47	0.74/0.93	
	Ref. 6	Target value	383	0.58	-	2.43/2.52
		Selected DSE sim.	380/386	0.56/0.57	1.07/1.13	
3.20 [3h12min]	DL predictions	Target value	741	0.55	1.00	2.04/2.23
		Selected DSE sim.	737/745	0.54/0.55	0.91/1.08	
	Ref. 6	Target value	656	0.69	-	3.05/3.52
		Selected DSE sim.	655/659	0.69	0.76/0.79	
4.75 [4h45min]	DL predictions	Target value	1473	0.68	0.79	3.39/3.71
		Selected DSE sim.	1470/1477	0.67/0.68	0.73/0.74	
	Ref. 6	Target value	806	0.76	-	4.01/4.09
		Selected DSE sim.	803/808	0.76/0.77	0.52/0.59	
6.08 [6h05min]	DL predictions	Target value	1212	0.73	0.70	3.55/3.78
		Selected DSE sim.	1213/1216	0.73	0.68/0.71	
	Ref. 6	Target value	1279	0.80	-	3.76/4.04
		Selected DSE sim.	1275/1284	0.80/0.81	0.47/0.52	

Table S4. DL-predicted structural descriptors n_{at}, χ_{Co}, and CS_{idx} from the *in-situ* GIWAXS data, at each Co deposition time, in the experiment with larger Ag seeds.

Time (h)	n_{at}	χ_{Co}	CS_{idx}
0.13	813	-0.03	-0.51
0.28	931	-0.02	-0.50
0.50	1100	-0.01	-0.47
0.73	1118	0.02	-0.41
0.95	1312	0.05	-0.39
1.18	1139	0.03	-0.42
1.40	1253	0.06	-0.37
1.62	1335	0.16	-0.10
1.85	1487	0.12	-0.23
2.07	1444	0.15	-0.10
2.30	1426	0.18	0.00
2.52	1487	0.23	0.14
2.75	1428	0.26	0.21
3.00	1538	0.24	0.19
3.25	1481	0.25	0.20
3.47	1628	0.30	0.37
3.68	1550	0.33	0.42
3.92	1553	0.33	0.44
4.13	1587	0.34	0.47
4.37	1556	0.37	0.52
4.58	1589	0.37	0.54
4.82	1807	0.37	0.52
5.03	1688	0.43	0.66
5.27	1800	0.40	0.62
5.48	1782	0.46	0.70
5.72	1749	0.43	0.65
5.93	1935	0.48	0.72

Table S5. In the GIWAXS experiment with larger Ag seeds, for each Co deposition time, the structural descriptors n_{at}, χ_{Co}, and CS_{idx} serving as "Target value" input to the search-match procedure described in the Supplementary Text are quoted. DL-predicted values from two alternative models (pink colored/white rows) are compared with the output parameter ranges identified by the search-march procedure and corresponding to the five most similar clusters. The corresponding minimum and maximum D_{Clark} values are reported in the last column. The two models were trained using patience 5 and 30 (labeled as *p5* and *p30*, respectively) and the same CNN architecture. For convenience, sample labels used in Figure 4 of the main text and in Figure S9, linked to this analysis, are also given in the first column.

**Time (h) [label]**	**Set**	**Type**	n_{at}	χ_{Co}	CS_{idx}	D_{Clark}
0.28 [17min]	*p5*	Target value	931	0.00	-0.50	5.60/6.08
		Selected DSE sim.	928/933	0.00/0.01	-0.55/-0.17	
	p30	Target value	1386	0.00	-0.37	5.59/6.33
		Selected DSE sim.	1384/1387	0.00/0.01	-0.46/-0.14	
0.95 [0h57min]	*p5*	Target value	1312	0.05	-0.39	3.36/4.85
		Selected DSE sim.	1311/1313	0.04/0.05	-0.46/-0.42	
	p30	Target value	1397	0.08	-0.32	3.36/3.77
		Selected DSE sim.	1395/1399	0.07/0.09	-0.45/-0.32	
1.62 [1h37min]	*p5*	Target value	1335	0.16	-0.10	3.29/3.52
		Selected DSE sim.	1333/1337	0.15-0.17	-0.21/-0.07	
	p30	Target value	1453	0.20	-0.03	3.21/3.34
		Selected DSE sim.	1452/1455	0.18-0.19	-0.12/0.09	
2.07 [2h04min]	*p5*	Target value	1444	0.15	-0.10	3.54/5.81
		Selected DSE sim.	1442/1446	0.15/0.16	-0.28/-0.03	
	p30	Target value	1453	0.15	-0.16	4.08/5.09
		Selected DSE sim.	1451/1455	0.15/0.16	-0.36/-0.15	
2.52 [2h31min]	*p5*	Target value	1487	0.23	0.14	2.84/3.53
		Selected DSE sim.	1484/1490	0.22/0.24	-0.01/0.26	
	p30	Target value	1417	0.25	0.14	3.05/3.35
		Selected DSE sim.	1416/1420	0.24/0.26	0.03/0.22	
2.57 [2h45min]	*p5*	Target value	1428	0.26	0.21	2.93/3.57
		Selected DSE sim.	1426/1430	0.25/0.26	0.17/0.45	
	p30	Target value	1464	0.31	0.37	3.23/3.63
		Selected DSE sim.	1463/1466	0.30/0.32	0.25/0.49	

6. Supplementary Figures

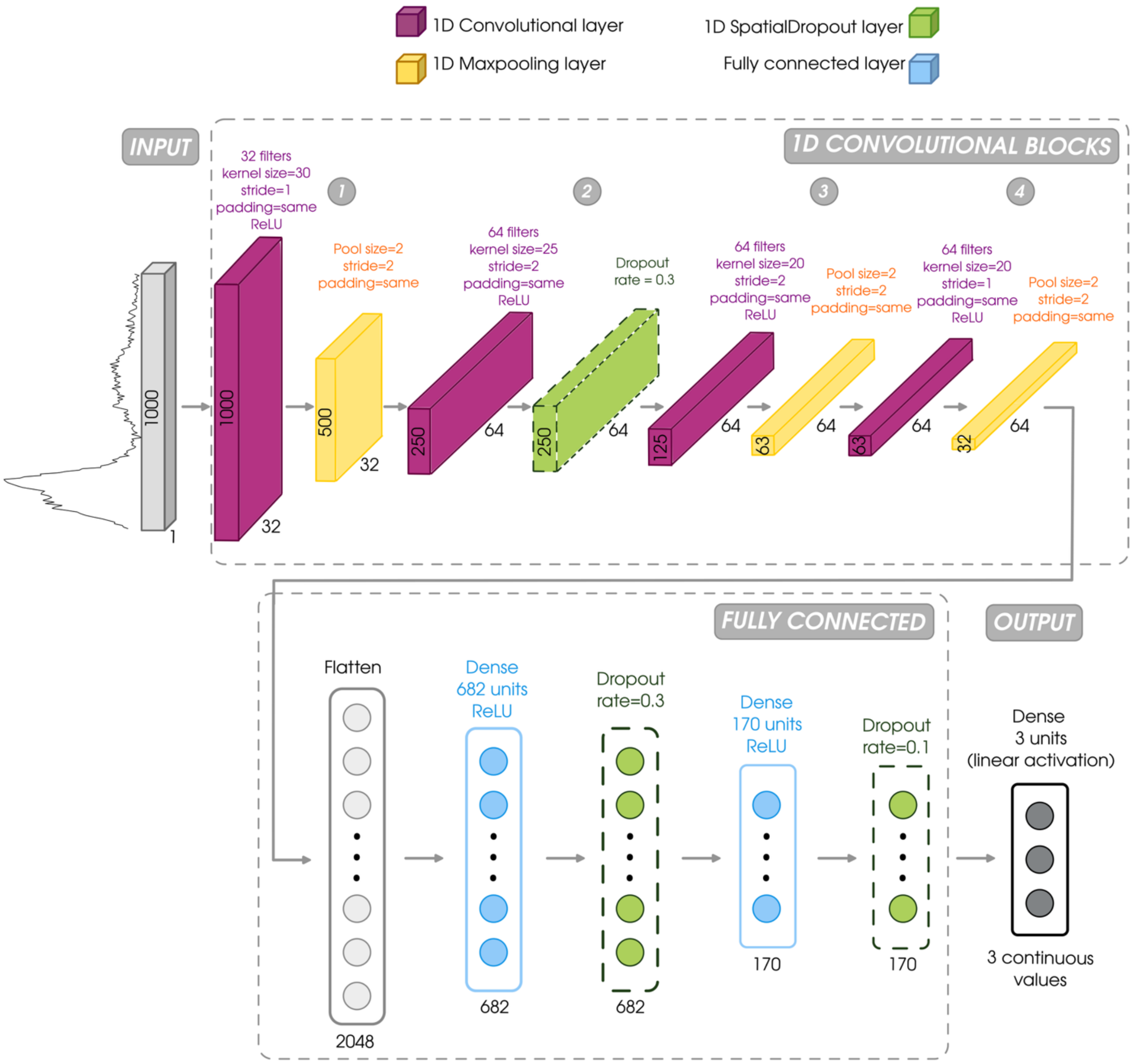

Figure S1. Schematics of the 1D neural network architecture developed in this work as detailed in the Supplementary Text. The output layer consists of three neurons, corresponding to the predicted structural descriptors (n_{at}, χ_{Co}, and CS_{idx}).

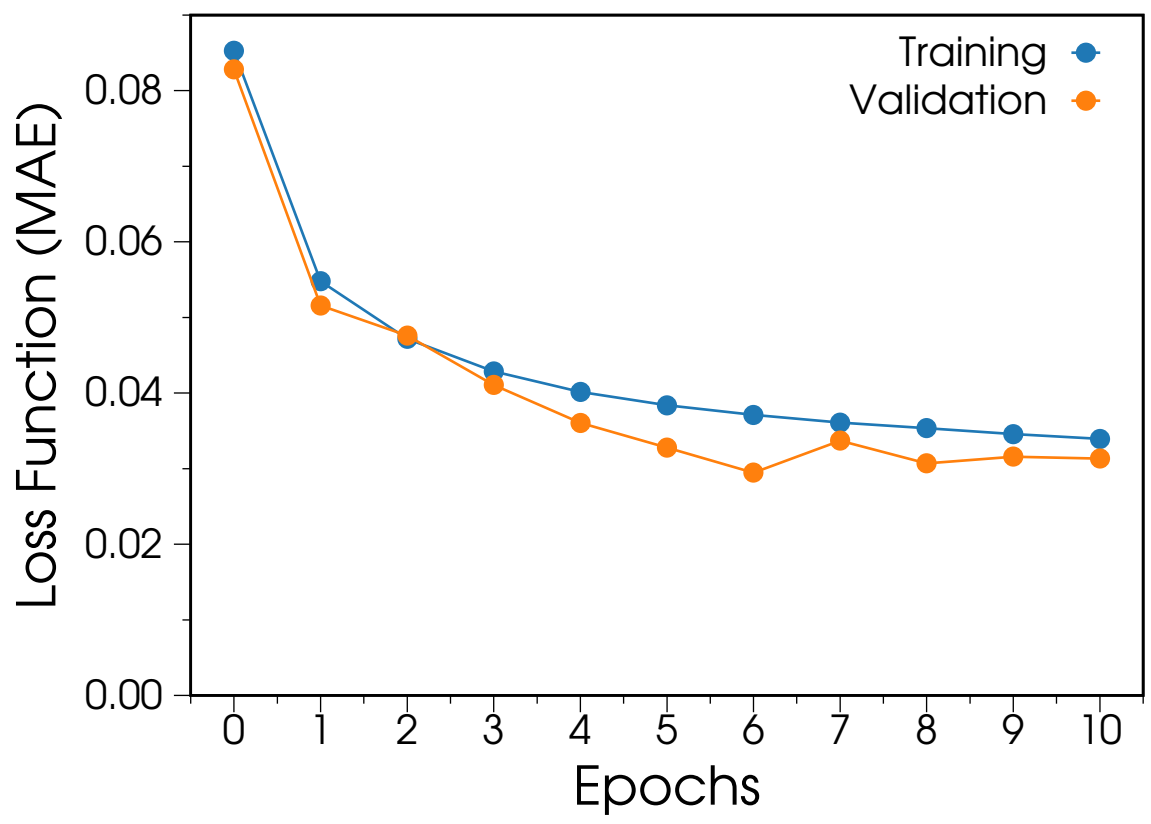

Figure S2. Loss function (MAE) across epochs for the training (blue line) and the validation set (10% of the training set, orange line) for the regressor. Due to the early stopping approach implemented (with patience 5), the best weights within the last 5 epochs are restored after convergence.

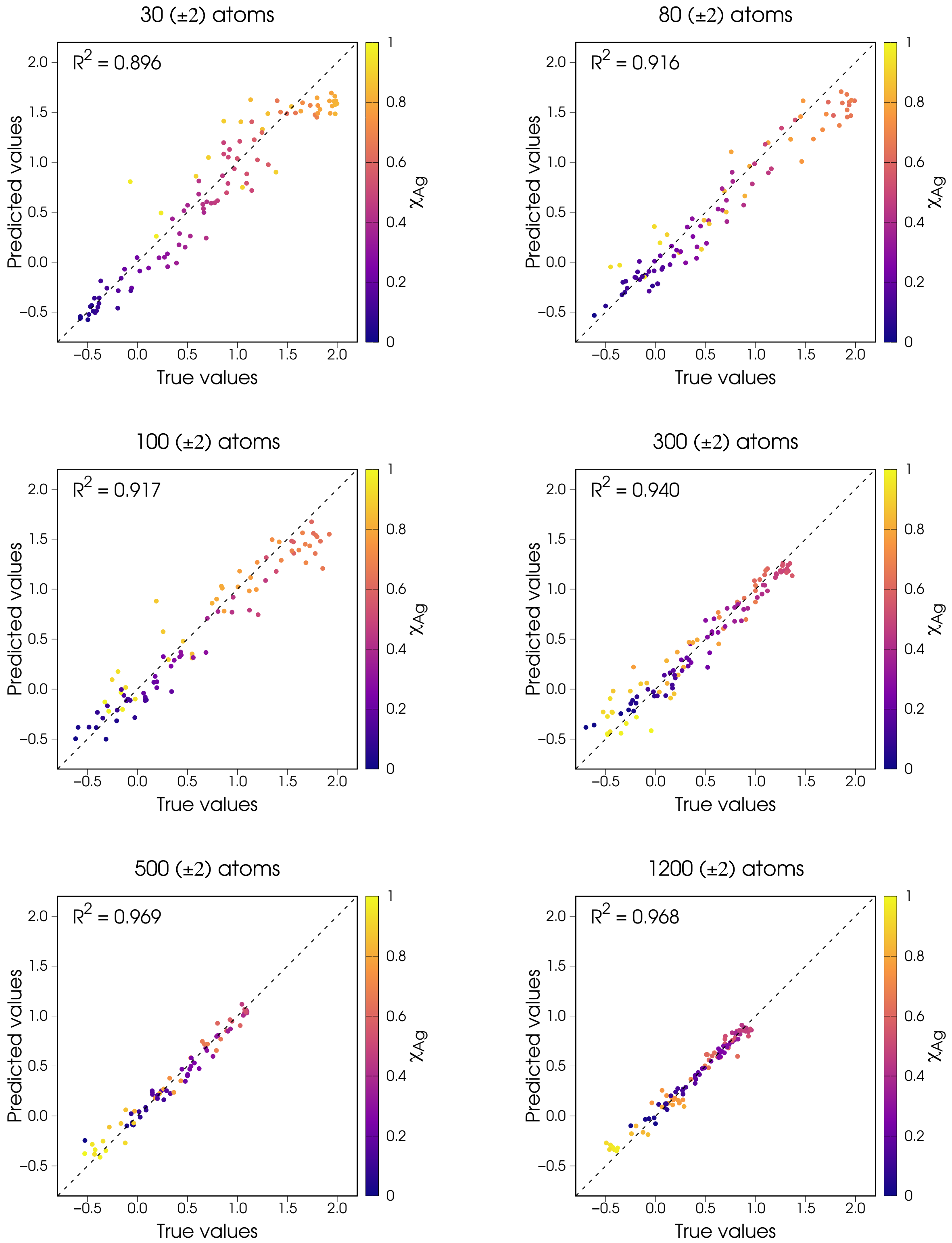

Figure S3. Parity plots for the CS_{idx} evaluated for clusters with selected n_{at}, spanning NPs with 30 atoms (~0.8 nm), 80 atoms (~1.3 nm), 100 atoms, (~1.3 nm), 300 atoms (~2.0 nm), 400 atoms (~2.2 nm), 500 atoms (~2.3 nm), and 1200 atoms (~3.1 nm). Scatter points are color-coded depending on χ_{Ag}, according to the colour bar on the right.

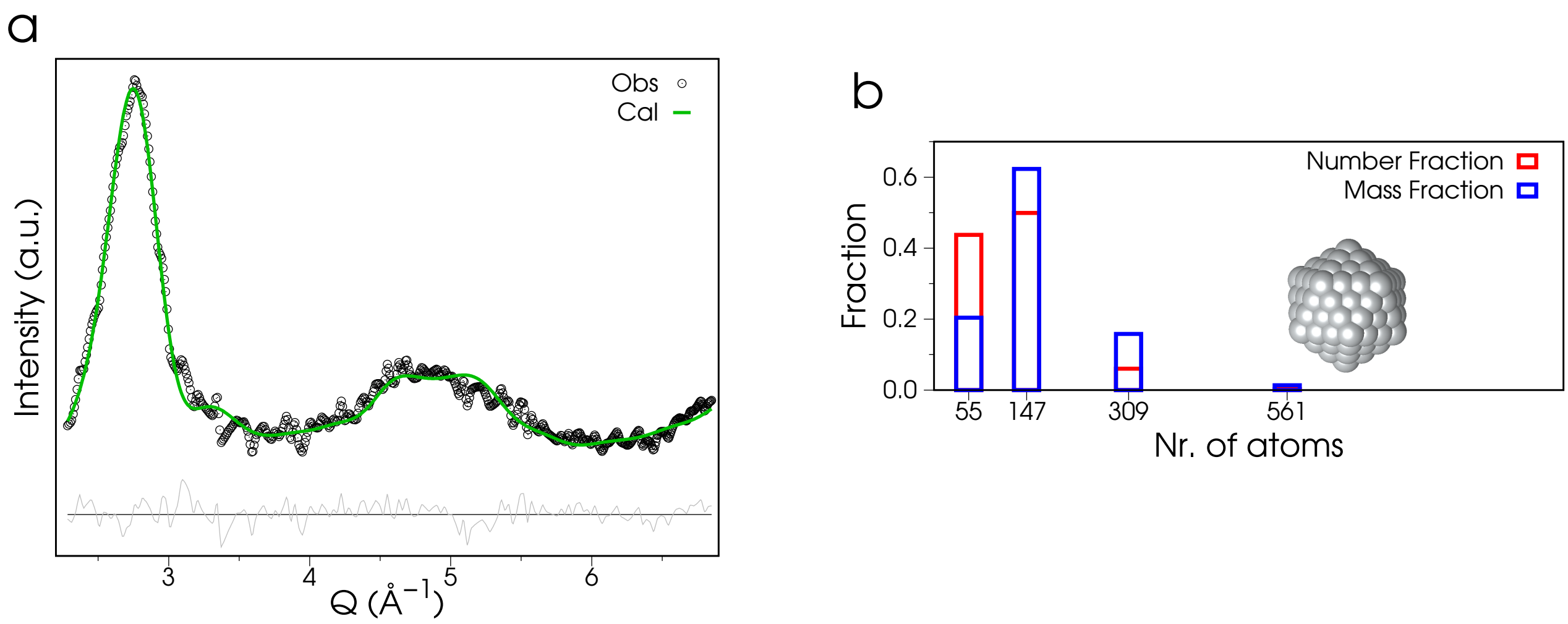

Figure S4. (a) GIWAXS data collected on pre-deposited Ag seeds (black dots, data subtracted of the substrate signal) and DSE best fit (green trace) obtained using icosahedral atomistic magic-number clusters of Ag NPs (shown in the inset), built according to the Mackay concentric shell model[7] developed in ref. [8], as detailed in the Supplementary text. (b) Number- and mass-weighted fractions *vs* number of atoms of icosahedral clusters, assuming a lognormal size distribution function, with mass-weighted average size (n_{at}= 160) and dispersion (σ = 96 atoms) refined against the experimental data. These correspond to an average diameter of the equivalent-volume sphere of 1.65 nm and σ = 0.34 nm.

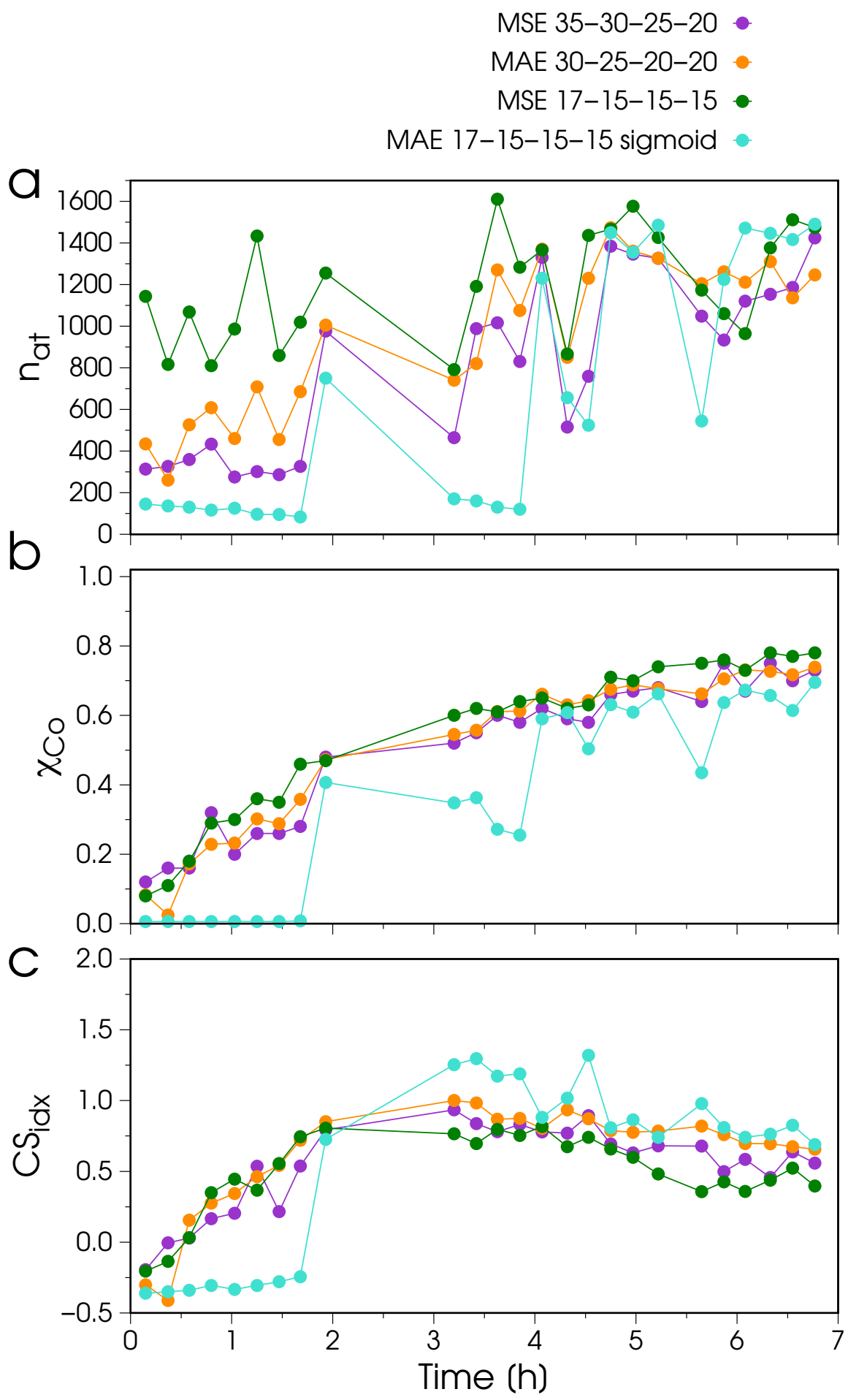

Figure S5. DL-predicted structural descriptors from the *in-situ* GIWAXS data: (a) total number of atoms n_{at}, (b) composition expressed as the Co fraction χ_{Co}, and (c) core-shell index CS_{idx}. The legend entries indicate the CNN architectures, where each string specifies the loss function (Mean Square Error, *MSE*, or Mean Absolute Error, *MAE*), kernel sizes, and activation function (*ReLU or Sigmoid)*, respectively. When no activation function is specified, the model employs *ReLU* activations for all layers followed by a linear output layer. For χ_{Co} (b) and CS_{idx} (c), the trends discussed in the main text are largely consistent across all tested models. A separate note is warranted for the model trained with a *sigmoid* activation function: because this activation saturates at its limits, it restricts the model ability to extrapolate and generally degrades predictive performance. In this case, the limitation is visible directly in the prediction trends, which remain nearly constant over a substantial portion of the initial growth period. This indicates that the model fails to capture the evolution of the X-ray patterns until the Co peak becomes clearly discernible and is no longer only a shoulder of the more intense Ag peak. Regarding the total number of atoms (n_{at}, panel a), notable oscillations are observed in the predicted values over the Co deposition time, along with significant discrepancies among models. These effects likely arise from the intrinsic difficulty of disentangling size- and defect-induced peak broadening in wide-angle X-ray scattering from ultrasmall, defective NPs such as those investigated here. The architecture reported in the main text corresponds to the model labelled MAE 30-25-20-20, which uses *ReLU* activations in all layers followed by a linear output layer.

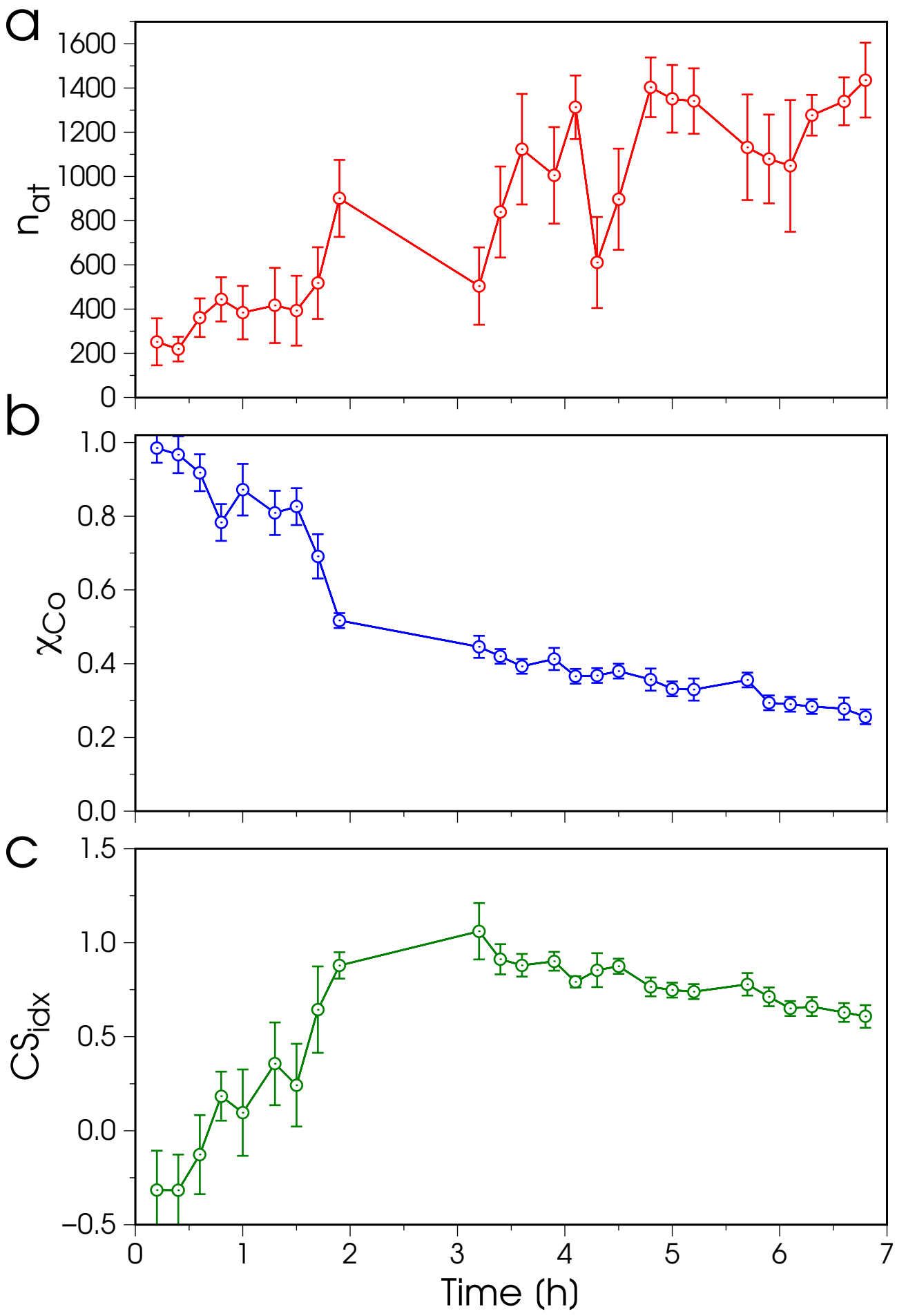

Figure S6. Predictions for (a) the total number of atoms n_{at}, (b) the Co fraction χ_{Co}, and (c) the core-shell index CS_{idx} obtained using the model discussed in the main text (architecture MAE 30-25-20-20 in Figure S4). The results shown here represent averages and standard deviations over 11 independent repetitions of the training procedure, all performed using the same network architecture but different random seeds for selecting the DSE simulations assigned to the training (80%) and testing (20%) sets. This approach provides an estimate of the variability in the predictions of the three structural descriptors for the selected CNN architecture.

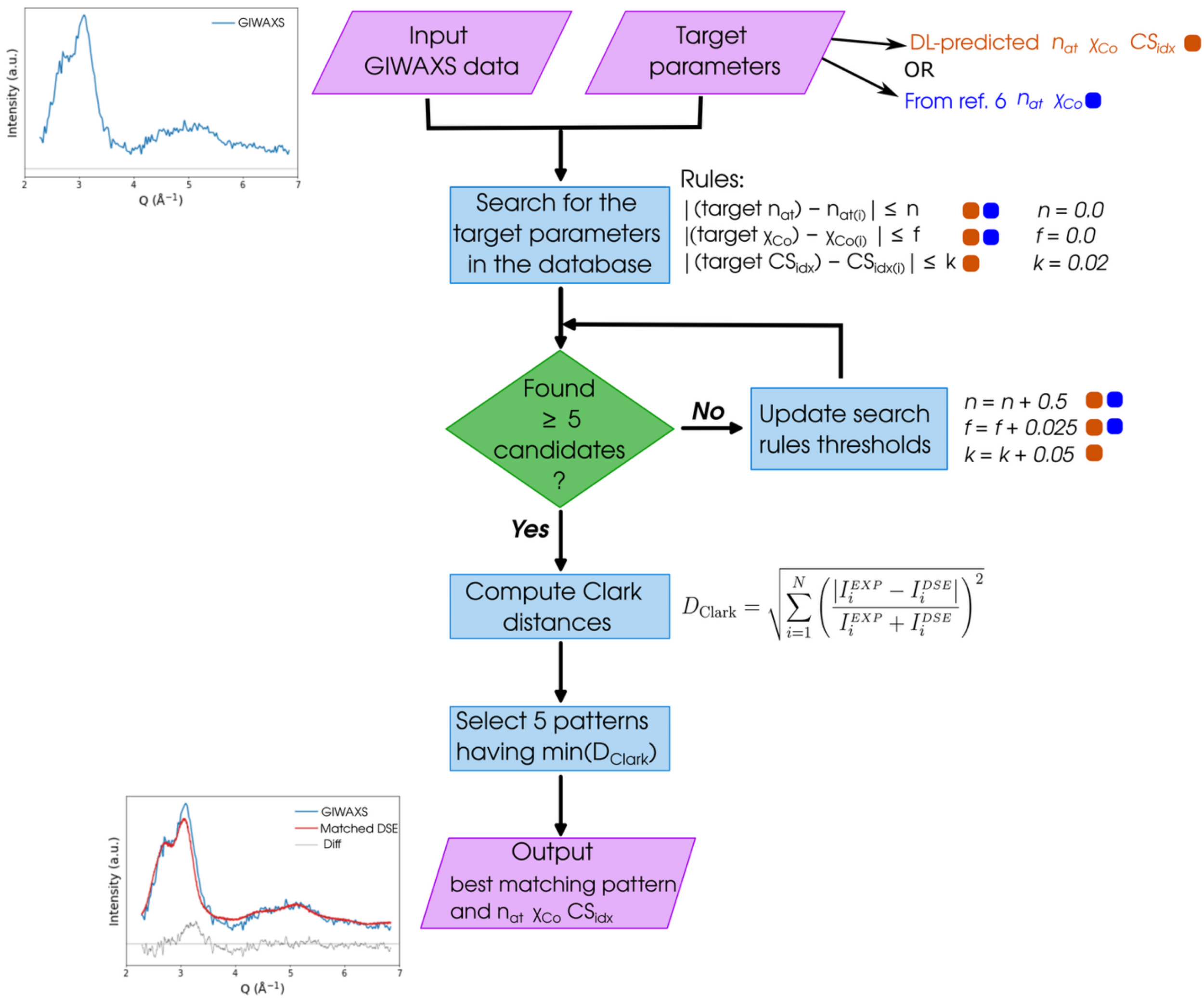

Figure S7. Schematic flowchart of the algorithm implemented to identify the cluster that best represents the information encoded in the experimental GIWAXS data. The output consists of the best-matching DSE simulation (lowest D_{Clark}) and related descriptors. Orange markers refer to the DL model predictions, blue symbols to the values reported in ref. 6.

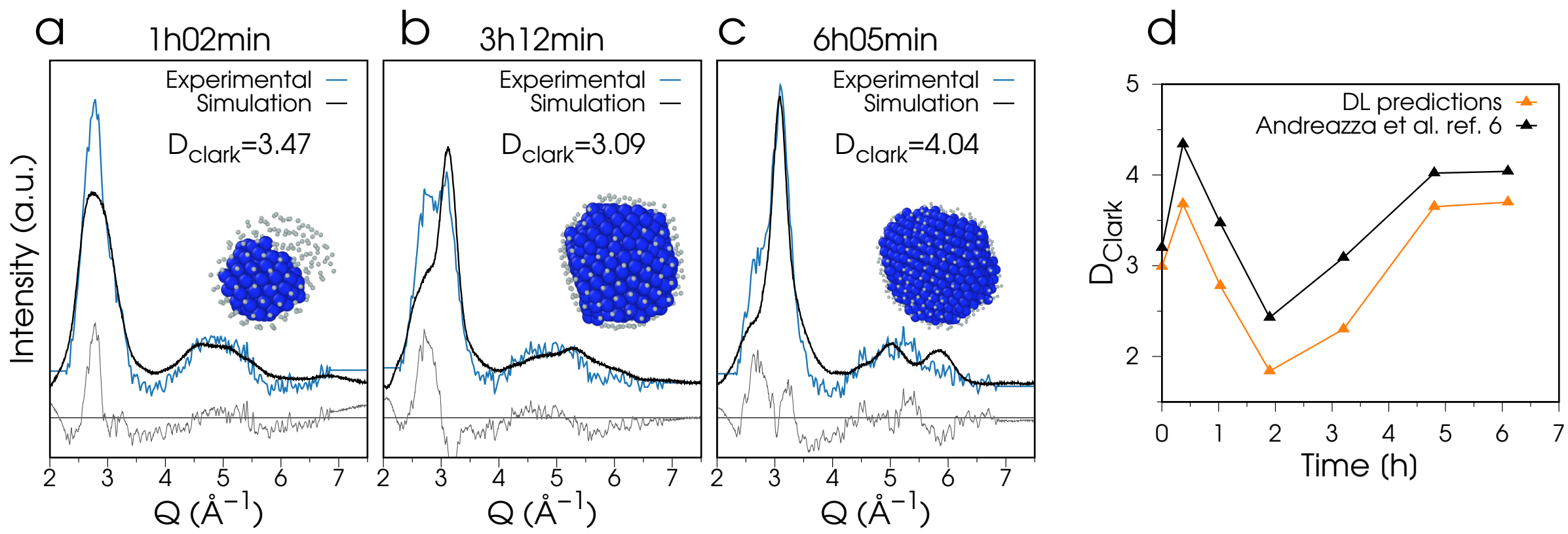

Figure S8. GIWAXS data (blue traces) for selected growth times and DSE simulations (black traces) encoding size and clusters composition from ref. 6 that best match the data, as identified by the search-match procedure. The selected growth times are: (a) an early Ag-rich stage at 1h02min, (b) an intermediate stage with comparable Ag and Co contents at 3h12min, and (c) a final Co-rich stage, at 6h05min. For each case, the corresponding D_{Clark} value and the MD atomistic model are shown (Ag in grey, Co in blue). (d) D_{Clark} *vs*. Co deposition time. Orange points refer to the CNN-predicted features (n_{at}, χ_{Co}, and CS_{idx} shown in Figure 3 of the main text), black points to from ref. 6. This comparison further demonstrates the overall poorer agreement with the experimental data obtained when assuming full incorporation of the nominal Co-deposited quantities into the final Ag-Co NPs (as per results shown in ref. 6).

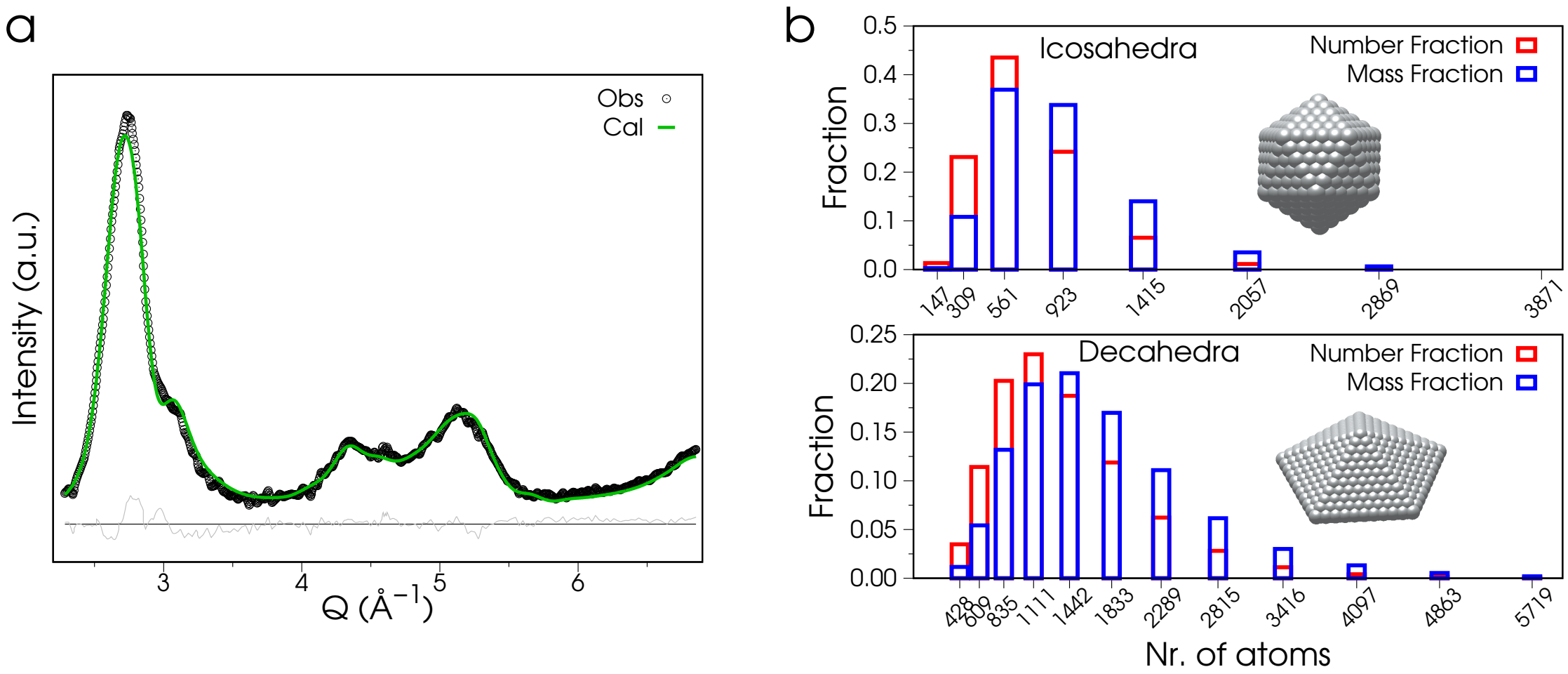

Figure S9. (a) GIWAXS data collected on larger Ag seeds (black dots, data subtracted of the substrate signal) and DSE best fit (green trace) obtained using a combination of icosahedral (85.5%) and decahedral (14.5%) atomistic magic-number clusters of Ag NPs (shown in the inset), built according to the Mackay concentric shell model[7] developed in ref. [8], as detailed in the Supplementary text. (b) Number- and mass-weighted fractions vs number of atoms of icosahedral (top) and decahedral (bottom) clusters, assuming a lognormal size distribution function. For the *icosahedral* population: mass-weighted average n_{at}= 842 and dispersion σ = 434 atoms (corresponding to an average diameter of the equivalent-volume sphere of 2.90 nm and σ = 0.48 nm). For the *decahedral* population: mass-weighted average n_{at}= 1605 and dispersion σ = 790 atoms (corresponding to an average diameter of the equivalent-volume sphere of 3.39 nm and σ = 0.56 nm).

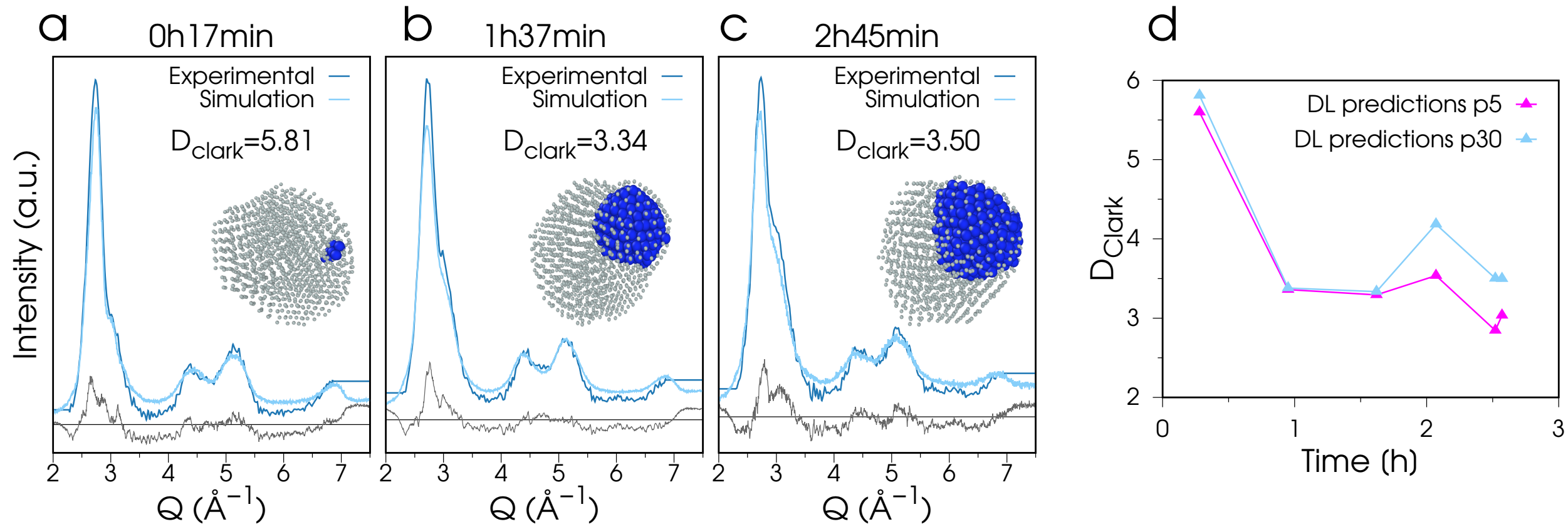

Figure S10. GIWAXS data of Ag-Co clusters (grown in larger Ag seeds, blue traces) for selected growth stages, and DSE simulations identified by the search-match procedure (light-blue lines). The corresponding CNN-predicted descriptors are obtained using a model trained with a patience value of 30. The selected growth stages are: (a) early Ag-rich stage at 17min, (b) 1h 37min, and (c) an intermediate stage at 2h 45min. For each stage, the corresponding D_{Clark} value is shown. The selected times are restricted to the first 3h of growth due to the upper limit in the cluster sizes available in the training database (1499 atoms, ~3.4 nm), while the CNN regressor can extrapolate beyond this limit and provides size predictions up to 1935 atoms (~3.7 nm) after 6 hours of experiments. (d) D_{Clark}values comparing the GIWAXS datasets with the DSE simulations generated from the CNN-predicted structural descriptors (n_{at}, χ_{Co}, and CS_{idx}) with patient 30 (p30) and 5 (p5); the corresponding DSE simulations with p5 are shown in Figure 4 of the main text.

(1) Dong, H.; Butler, K. T.; Matras, D.; Price, S. W. T.; Odarchenko, Y.; Khatry, R.; Thompson, A.; Middelkoop, V.; Jacques, S. D. M.; Beale, A. M.; Vamvakeros, A. A Deep Convolutional Neural Network for Real-Time Full Profile Analysis of Big Powder Diffraction Data. *npj Comput Mater* **2021**, *7*, 74.
(2) Abadi, M.; Agarwal, A.; Barham, P.; Brevdo, E.; Chen, Z.; Citro, C.; Corrado, G. S.; Davis, A.; Dean, J.; Devin, M.; Ghemawat, S.; Goodfellow, I.; Harp, A.; Irving, G.; Isard, M.; Jia, Y.; Jozefowicz, R.; Kaiser, L.; Kudlur, M.; Levenberg, J.; Mane, D.; Monga, R.; Moore, S.; Murray, D.; Olah, C.; Schuster, M.; Shlens, J.; Steiner, B.; Sutskever, I.; Talwar, K.; Tucker, P.; Vanhoucke, V.; Vasudevan, V.; Viegas, F.; Vinyals, O.; Warden, P.; Wattenberg, M.; Wicke, M.; Yu, Y.; Zheng, X. TensorFlow: Large-Scale Machine Learning on Heterogeneous Distributed Systems. Mar. 16, 2016. Http://Arxiv .Org/Abs/1603.04467. arXiv March 16, 2016. http://arxiv.org/abs/1603.04467 (accessed 2026-03-14).
(3) TensorFlow Documentation TensorFlow. Https://Www.Tensorflo w.Org/Api_docs/Python/Tf.
(4) Keras Documentation Https://Keras.Io/Api/.
(5) Park, W. B.; Chung, J.; Jung, J.; Sohn, K.; Singh, S. P.; Pyo, M.; Shin, N.; Sohn, K.-S. Classification of Crystal Structure Using a Convolutional Neural Network. *IUCrJ* **2017**, *4*, 486–494.
(6) Andreazza, P.; Lemoine, A.; Coati, A.; Nelli, D.; Ferrando, R.; Garreau, Y.; Creuze, J.; Andreazza-Vignolle, C. From Metastability to Equilibrium during the Sequential Growth of Co–Ag Supported Clusters: A Real-Time Investigation. *Nanoscale* **2021**, *13*, 6096–6104.
(7) Mackay, A. L. A Dense Non-Crystallographic Packing of Equal Spheres. *Acta Cryst.* **1962**, *15*, 916–918.
(8) Cervellino, A.; Giannini, C.; Guagliardi, A. Determination of Nanoparticle Structure Type, Size and Strain Distribution from X-Ray Data for Monatomic f.c.c.-Derived Non-Crystallographic Nanoclusters. *J. Appl. Crystallogr.* **2003**, *26*, 1148–1158.
(9) S, S.; K, A.; Dzubinska, A.; Reiffers, M.; R, N. Systematic Investigations on the Magnetic Properties of Moderate Heavy Fermion $CeAg_{0.68}Si_{1.32}$ Alloy. *Physica B Condens. Matter* **2019**, *575*, 411679.
(10) Hull, A. W. X-Ray Crystal Analysis of Thirteen Common Metals. *Phys. Rev.* **1921**, *17*, 571–588.
(11) Hernández-Rivera, E.; Coleman, S. P.; Tschopp, M. A. Using Similarity Metrics to Quantify Differences in High-Throughput Data Sets: Application to X-Ray Diffraction Patterns. *ACS Comb. Sci.* **2017**, *19*, 25–36.
(12) Cervellino, A.; Frison, R.; Bertolotti, F.; Guagliardi, A. *DEBUSSY* 2.0 : The New Release of a Debye User System for Nanocrystalline and/or Disordered Materials. *J. Appl. Crystallogr.* **2015**, *48*, 2026–2032.
(13) Martin, T. P. From Atoms to Solids. *Solid State Ionics* **2000**, *131*, 3–12.
(14) Cervellino, A.; Giannini, C.; Guagliardi, A. On the Efficient Evaluation of Fourier Patterns for Nanoparticles and Clusters. *J. Comput. Chem.* **2006**, *27*, 995–1008.
